# Supplementary material for: What is the lived experience of anxiety for people with Parkinson’s? A phenomenological study
Source: PLoS One. 2021 Apr 8;16(4):e0249390. doi: 10.1371/journal.pone.0249390 (PMC8031398; doi:10.1371/journal.pone.0249390)
Supplement: S1 File — (DOCX) [file pone.0249390.s001.docx]

**Interview schedule**

**Opening**

My name is Chris Lovegrove and I’m doing this research with Plymouth University. I would like to ask you some questions about your background, your condition, some experiences you have had, and about you. You do not have to take part if you do not want to.

I hope to use this information to help develop more anxiety interventions specific to people with Parkinson’s. The interview should take about one hour, but may be a little shorter or longer. Please feel free to ask for any breaks that you might need during the interview. Are you happy to continue?

Transition: Let me begin by asking you some questions about where you live and your family. Are you still happy to proceed?

**Contextualisation**

- Can you tell me about yourself?
- Please can you tell me about your diagnosis with Parkinson’s.

*Prompts as needed*

**Apprehending the phenomenon**

- Please can you tell me about your typical day?
- What is your experience of anxiety?
- How does anxiety affect you?

*Prompts as needed*

**Clarifying the phenomenon**

- Can you describe how anxiety makes you feel?
- How do you react to anxiety?

*Prompts as needed*

**Closing**

I really appreciate the time you took for this interview. Is there anything else you think would be helpful for me to know? *(Time for answers)*

Do you have any other questions for me? *(Time for answers)*

Would you like a summary of the findings? *(Time for answers)*

Thank you, I have all the information I need. I will now stop the recording. Thank you again. *(Recording stops, start debrief)*
