# Supplementary material for: What is the lived experience of anxiety for people with Parkinson’s? A phenomenological study
Source: PLoS One. 2021 Apr 8;16(4):e0249390. doi: 10.1371/journal.pone.0249390 (PMC8031398; doi:10.1371/journal.pone.0249390)
Supplement: S2 File — (DOCX) [file pone.0249390.s002.docx]

**CL- My name is Chris Lovegrove and I’m doing this research with Plymouth University. I would like to ask you some questions about you background, your condition, some experiences you’ve had and about you. You do not have to take part if you do not want to. I hope to use this information er to develop more anxiety interventions specific to people with Parkinson’s. The interview should take about one hour but it may be a little shorter or longer. Please feel free to ask for any breaks that you may need during the interview. Are you happy to continue?**

Alan**-** Yes I am.

**CL- SO let me begin by asking you some questions about where you live and your family. Are you still happy to go ahead?**

Alan**-** Yes I am of course. Yes.

**CL- Erm SO can you tell me a little about yourself?**

Alan**-**  Yes my name is <name>. My surname is <name> spelt **** in Salisbury in Wiltshire erm and I’m willing participant in this survey. I am currently just over 60 years old and before I moved to Salisbury I lived in Norfolk and I moved down here because there was family here and then they moved and I was left here. I am married I don’t have any immediate children with my wife at present but I do have children from my first and I’ve worked basically all my life in construction work I’m a construction manager. Site manager. I’ve always considered myself to be fit and well which I am. And I’ve always gone through my life and and generally been ok and then I had the bombshell about I think it’s three and a half years ago now that I was diagnosed with Parkinson’s. Er that started with I noticed that a seminar that I went to on health and safety that my right hand which is hand that I predominantly use er, started to just twitch with the thumb and the forefinger. And I had that I had difficulty with my writing, er it I would be writing all day, for a couple of paragraphs and then the writing would be more difficult to achieve and get smaller HRRRMMM <clears throat>. I didn’t take any notice of it I just thought it was something ha.

**CL- Hm yes, yes.**

Alan **-** I didn’t really understand what it was all about. And in fact I related the twitch to my thumb and my forefinger to a muscle strain in shoulder due to pulling a big door together on the Friday night when I was leaving my site. In Southampton actually. And then my wife said “Yo-y-you know this twitch what’s going on with your thumb and forefinger” and I said it’s nothing it’s probably a trapped nerve and I, I , I didn’t really pay any attention for a long while and then it started getting worse and I decided to go to the doctor and the information on that day was as follows. “Good afternoon sir, come in to the surgery. Er what is the problem” I have a twitch in my thumb and forefinger and I’ve had a little difficulty with my handwriting. Within about five minutes stand up, walk over there lift your hand up, do this with your fingers. Ah rotate our fingers through each other if that makes sense.”

**CL- Hmm.**

Alan **-** Touch. Tip touch. Err he said “you’ve got Parkinson’s” just like that and I said pheew no! And he said “well it’s either that or you’ve got a brain tumour” and with that my world collapsed within about thirty seconds.

**CL- Hmmm**

Alan **-** And that he said “I will refer you to the hospital” which is exactly went to have an appointment with Dr <name> which is the neuro consultant at Salisbury district hospital and <name> the movement disorder nurse. Ah two great guys. They said you’ve got what’s know as young persons onset resting tremor. And I still couldn’t take in what was happening and immediately the picture immediately on discussing with them the picture I had in my mind which is portrayed sadly, throughout the Parkinson’s brochures and everything, cos I was advised to read and er and a find out what the condition means what you can prepare for and it’s it you get the picture of a poor old man walking down the road snivelling, dribbling, smoking or looking very forlorn and I was depressed for about three months after that b’cos every piece of information I read there was nothing really positive. And even if you read I can’t think of the movie star Michael Fox is it?

**CL- Yes that’s right, Michael J Fox.**

Alan **-**Michael J Fox, some of the stuff I read in his book was detrimental to the information for me at that time. I just felt my whole world had come apart because I’d always been fit and strong n whilst I’m 60 years old, er a lot of people wouldn’t consider me to be that. I’m healthy. Ahh so anyway, I, I immediately hit rock bottom and it took me quite a long time to get used to the fact that I had, I had Parkinson’s disease diagnosed. Er I haven’t had anything except the tremor that gets worse. I was put on a regime of drugs at the hospital, various ones in the three and a half years I’ve had, whilst I’ve been taking um, er what shall we say meetings with the consultant sorry I couldn’t think what, what. Since I’ve been having appointments with the consultant. Ahhnd various drugs haven’t actually done nothing at all.

**CL- Right.**

Alan **-** At the moment I take sastravi. Er with levodopa and two other constiuents which are in there. It’s not a mega high dose but it’s quite reasonable but it hasn’t done anything no’isable er for my tremor. And I’ve been dealing with things generally since then. My, my current position is that on the second of February I’m about I’m about to go to Bristol hospital Sunnymeads or Runnymeads I think it’s called. Er ah under the auspices of Professor <name> and I’m gonna I’ve been selected for deep brain stimulation.

**CL- Ok.**

Alan **-** Which will hopefull uh deal with the tremor it won’t cure the Parkinson’s I’m aware of that. But firstly I’m going for my scans on the second and the tests for the anaesthetistto make sure I’m ok for what type I need and how long I need to be put out. They’re going to map my brain so they understand where all the components are, the blood vessels the point where they need to put the stimulator in. And hopefully that will help me with my tremor because it is getting worse and, and that in itself makes you anxious. The the thought of going and having placed into your head its not like erm taking pills for the flu or havin operation on your arm or your foot or something it’s really in there where everything counts. So that brings its own set of challenges itself.

**CL- Hmm**

Alan **-** Aaand yeah I’ve had I’ve had times where I’ve felt depressed. And I haven’t taken medication I mean I’ve always been one for I, I’ve always been a one to make sure I don’t look down that avenue I don’t suffer with depression or haven’t had depression. Even though I was down in a bad place when I started I managed to pull myself through without having to I went to PD Warrior.

**CL- Yes I’ve heard of that.**

Alan **-** Which is very beneficial, a because it’s six guys we started out with and we are close knit family if you want to call it that and that’s good camaraderie and that’s good for your own wellbeing and physical exercise has been proven in Parkinson’s to to, to help and supress the anxiety things and also to keep you fitter longer so that the disease doesn’t take over your skeletal frame if that’s a way of explaining it.

**CL- Yeah that makes sense.**

Alan **-** Erm and… and I do that I walk and I do a little gym work to try and keep fit. For instance we’ve just been on a short break over the Christmas period and we walked on average about 13 kilometres a day. Sometimes a little more.

**CL- Wow, getting the activity in.**

Alan **-** Getting the activity in. Erm which helps and it does help definitely if you are outside and you’re doing somethin’. I find that now because I’m home that, a because several things ‘ve happened and I’ve had to plan them in but I find that if your home alone and you’re not doing y’daily, daily routines like going to work that makes you feel a little subdued and that you start thinking about things and you can be walking around and you fixate on things like you must tidy up, you must get cleared round. Little things like that they become more of an issue than say erm, washing up laid up and becomes “uh no why can’t we keep this more tidy”. Little things like that and if you don’t get to deal with them it becomes more, you become anxious that you’re not actually achieving anything. Does that make sense?

**CL- Yeah, that’s very clear, thank you.**

Alan **-** I-it gives you, if y’not don’t complete tasks which keeps you busy which takes your mind off, off, off the problems you might have, and of course your not doing things as you would’ve done all your working life. There’s a big change. I’m physically able to do work. But. You find yourself concentrating moreso that your not making mistakes because you’ve got a shakey hand, and your not as competitive as you were or as strong as you were and it’s also difficult to find people to talk to about it because everybody, everybody doesn’t want to hear your, y’know how your feeling everyday particularly for men I think it’s ah that’s about the sort of thing you feel. And its its always a constant thing in the back of your mind what’s going to happen in the future which also makes you think about things in a different light as well. It causes you to think am I goin ta be mobile, whats it going to mean for my wife cos I have a younger wife than I am, and what that all means with your own personal affairs there and what it’s going to mean for her, is he going to be a carer, so there’s a wealth of things that come to mind that all make you feel. Yeah I think the word is concerned and anxious for those reasons, does that make sense?

**CL- Absolutely.**

Alan **-** Erm. It would be easy to go to the doctor and say about these things and you can’t really, I don’t want to waste his time and I don’t think many people would. And you can always be given tablets for things but that that isn’t the answer it’s about being able to overcome sometimes that you even get the sense, you feel useless. Y-y-you tend to feel like, your not the same person any more, your not doing the same things, i-it gets you down whereas it I mean my hobby for arguments sake is fly fishin’. I love. I had a complete meltdown. I went fishin’ and I was dreadin’ the, the business of tying the fly on the tibbit.

**CL- It’s quite fiddly isn’t it.**

Alan **-** It’s fiddly and I couldn’t achieve that hardly, and I, and it was a little bit cold and I’ve come to realise that in that weather it doesn’t help the Parkinson’s tremor so there’s a little bit of a learning curve there. And I had a little bit of a breakdown on the, on the river bank. Luckily I was on my own and I had a bit of a temper because I was on my own because I couldn’t. I wasn’t achieving what I could always achieve easily so there’s all those types of feelings that you have in your, in your, in your enjoyment items in life and your hobbies, and you also, I also feel more fatigued through the Parkinson’s which is a frustration if you’re a fit chap, you find when sometimes I get up and have a walk in the morning, with my colleague up the route whose also got Parkinson’s, and I can come back and feel ok and if I come back sit down and have a cup of tea I’ll feel tired and, I’ll just need to have 20 minutes nap and little things like that and you think ‘blimey is this what’s going on’ y know. I-I can’t put my finger on it exactly the words to describe how your feeling i-its erm, yeah. Do you understand what I mean?

**CL- Thank you <name>, thank you. I was wondering if we, you mentioned about your diagnosis and how tht was quite a big moment…**

Alan **-**It was huge.

**CL-…I was wondering if you could tell me a little bit more about that event, the diagnosis process and how it made you feel.**

Alan **-** Like I say I-I went to the doctor, cos like all a lot of people things didn’t concern me all that much I thought it was a strain o-or a twitch o-or something was catching in there so it would be ok. My wife said she thought, because she’s a doctor of dentistry, erm she thought there was something on which is why she kept prodding me to go there. The way the diagnosis came about was point of fact if y’like. With no emotion attached to it, with no… it seemed like someone read it out of the newspaper. Err sorry Mr, err today on the 20^th^ of so and so, Mr <name> has got Parkinson’s. Oh, it may not be Parkinson’s it could be a brain tumour. Erm I’m sorry to have to tell you but we’ll send you to the hospital. Thank you very much. Goodbye. And it was just like that. And I can remember just, when I came out from that meeting and got myself into a private place alone I just fell to pieces. Because automatically the picture of the poor old soul walking up the road snivelling drivelling and dragging his legs behind him is exactly what you think is going to happen to you. You hear about it and even in life people joke about it, they say, he’s shaking or something have you got Parkinson’s you’re not paying attention and all the rest of it. Prior to that I realised my friend up the road had had it, and I though uh dear have I got this to bear? Is this now coming upon me. And particularly if you’re fit, open minded, diligent with your work and active it just, your like a rabbit in the headlights. I joined this little group of young Parkinson’s sufferers, that was a good thing for me and they said to me when I first went to join them as a group erm, they could tell I was anxious and I didn’t know what to expect and I was asking all sorts of questions and, I could see the varieties of Parkinson’s staring me in the face by being with those guys. Some were a little better, some were a little worse, and it’s coming to terms with how you deal with that, and what people you have around to try and help you, and yeah probably three months I just find myself at any time just thinking about things and just breaking down and crying. And you can’t, I-I’ve now discovered this is can be part of the Parkinson’s issue it affects your emotional being and I’ve noticed since I’ve been diagnosed as well I’m more susceptible to issues that make you emotional, like for arguments sake erm, a piece of music that will have a nice meaning o-or deep thoughts things like this, or someone will lose someone and you’ll think of it and you’ll feel upset, you can’t help it. So you you, it, it makes you think about the Parkinson’s thin, where your off too, and h-how how do you actually deal with it, how can I actually get through this, and I find I try and find I must always be busy. And for one reason if I’m not busy using my hands, or doing something. Then the tremor annoys me, because the pills don’t work, and then you find yourself getting more anxious and then, that feeds the tremor. It’s emotionally draining it it’s also physically draining depending by the end of the week what sort of a week you’ve had, you can just feel whacked by Friday lunchtime. And its it’s a cycle like that more or less everyday.

**CL- Ok. That leads on nicely to the next question would you be able to tell me what your typical day is like? What your daily routine is like?**

Alan **-** Well before I was diagnosed with Parkinson’s I was construction site manager so I would be up for work at anything from half past five to be at work ready for half seven wherever my site was so I was always full out busy, every single day and it was tiring, very stressful job. Extremely stressful job. And when I had the diagnosis and I saw the consultant, h-he said I should consider the way forward because it’s life changing, not life threatening as such. So I made the decision to come out of construction management and I though what, well I couldn’t work properly in the first months anyway because I just didn’t know where I was, down in the dumps. SO I decided the only thing to do for me was to go back to my routes because I’m a tradesmen as well, as well as having my construction management degree so I went back I bought myself an old van, got some tools, and set about doing little jobs for people, which I still do. But, since erm, since a little while ago, since the possibilities of having to go into the hospital which they advise me might be anytime in the early part of December or mid-December, to have the scans and everything as a preparation for the operation in the New Year, then I thought to myself, well I need to obviously do something so I set about converting my outhouse into a shower and a toilet so if in time my disease gets worse and I can’t get up the stairs to use the bathroom at least I can have a shower, it doesn’t force me out of my house. Or at least it doesn’t force me to have big construction conversions to enable me to be accommodated if I’m wheelchair bound for arguments sake. So, I’ve been doing work on and off and with the tradesman I’ve had help me here, yes I’ve been doing small jobs and yes you sometimes become frustrated with them and you, you , you can be tempted not to attempt things as you become worried not to make mistakes. It’s like you’re a write and all your writing is terrible and, it might not make sense because you can’t concentrate so you think I’ll leave it today and then today becomes another day. So you must still attempt tasks it gives you purpose to be dealing with stuff. You definitely need to have exercise but in the construction world it’s a double-edged sword because, erm, you need to think a lot, to be able to complete the task to a good standard but it’s also physically demand which I’ve found is good for me.

**CL- Right.**

Alan **-** So, because it keeps me occupied it uses my body, ok so as a older guy it’s a little more difficult nowadays as opposed to where I was and it’s a retrospective move I’ve gone backwards in my career, not forwards, and I was on a good path. But because I can’t use the computer very well, as you can see my hand is shaking, it gives your confidence a kick, and that irritates you as well because you find you’re pressing the buttons wrong or scrolling and eradicating information. That’s part of the reason I decided not to carry on with the management because if your dealing with drawings and such and and computerised requirements dealing with documents and details it’s a pain. SO I try to keep myself busy by doing small jobs basically. I’m very lucky to have a supportive wife. And, yeah she’s very helpful, she does care for me, she makes sure I’ve got some things ready and makes sure I’m taking my medication because another thing when you’re busy and you’ve got Parkinson’s, even if you have to only take one tablet three or four times a day you find yourself getting engrossed in what your doing and find yourself inadvertently thinking about the Parkinson’s issues whilst you’re busy and you forgot to take your medication, and then you think oh did I forget to take my tablet at twelve o clock or whatever. Erm and did I actually do that and you find yourself getting engrossed in things. Y-you get sidetracked does that make sense?

**CL- That makes sense.**

Alan **-**  You’re thinking about doing your work and the Parkinson’s things kick in and you think yeah I wish my hand would stop shakin’ and everything and so time goes through and you forget that you, ooh I should’ve took my medication.

**CL- And when you have that realisation that you’ve forgotten something…**

Alan **-** It makes you more anxious, it makes you more urgent you think ah hell I should’ve done that I need to get there and do that now you could be busy doing something you need to concentrate and because the issues been discovered, because you’ve discovered you should’ve done something you’ll stop what you’re doing and go and do it. And that, that, you can feel anxious have I left anything out whilst I go and do that do you see what I mean?

**CL- Yeah I see what you mean.**

Alan **-** It’s a funny, it’s a funny, it’s a, it’s a very difficult thing to describe because it leaves you feeling sometimes jittery, sometimes you can’t explain your feelings or you won’t be able to concentrate or you won’t be able to listen if someones talkin’ to y’ as your minds thinkin’ I must do this this this. Does that make sense?

**CL- That makes sense, yeah.**

Alan **-** So you’re trying , you’re trying, you’re trying to keep on an even keel er and in my circumstance I’m trying to think what the way forward is. I’m trying to think about earning money, I’m trying to think about my wife is ok and that I can afford to pay the mortgage which is all these things going around everyday. I think it’s not good, definitely not good to be a lone at home too long for anybody. Without being, without having contact with other people.

**CL- Could you explain a little more about that?**

Alan **-** Because you start, you start getting in depth, or you start maudlin I think is the word, you can feel a bit down about things. You can think, oh hell. You don’t really, you get lazy in the mind. You become, you think about things which bring on an anxious feeling. I can’t explain it. It’s a…. yeah it’s very difficult to explain the feeling. It’s like being in some sort of a, some sort of a position you can feel irritated, you can feel agitated, you can feel though your not concentrating on one thing and then you will concentrate on something else then you’ll remember something else you shoulda done, that will make you feel ah heel I shoulda done that why didn’t I phone so and so. And there’s all little thing like that which culminate in sometimes you just feel cheesed off. You know I mean, I don’t, I don’t think that I’m the sort of person that’s generally like that I’m forward thinking so I’m trying to give you an insight into, it-it can be a bit muddling.

**CL- Muddling. Ok that leads on to quite nicely to the next question. What is your experience of anxiety living with Parkinson’s?**

Alan **-** My experience of it is, it can leave you nervous, it can make you nervous. It can make you very concerned about issues that might not be so difficult to deal with but they become a problem more than they really are. If you stand back and analyse it and sometimes I’ll do that I’ll say to myself, right do this first then do that then we’ll go from there, particularly if you’re not with your regular work crew you have to work things out for yourself how to deal with certain things. How to bat off of someone for arguments sake. You have to find certain ways and means of dealing with them off your own back. Are you making the right decision? Sometimes you can doubt yourself and question whether you did actually measure something twice to check I’ll measure it again. Whereas normally you may say, I’ve measured that, it logs in your mind, and you know you feel safe that you’ve done that. Because of the Parkinson’s it gives you an element of doubt. It can give you an element of doubt and an urgence to make sure that you’ve checked it again. T-to make sure that you haven’t made a mistake, or that you should’ve gone to the shop or something, it’s a very very difficult thing to discuss because it varies.

**CL- Ok. Does it vary day to day, or is it something a bit more than that?**

Alan **-** Yeah so if I’m out doing something on a normal day say a days run and I’m concentrating on my work and it’s a good day you can forget about things, and it a, it brings you a degree of pleasure out of your normal work again because your not thinking about the Parkinson’s again, and the Parkinson’s isn’t, isn’t in control if you like. That’s a good way of saying it. Because the Parkinson’s is trying to take control because of the loss of dopamine. So. And the other thing of course if you have Parkinson’s a lot of the other guys we’ve all said together you find the urgency to go for a pee all the time. And that in itself if you’re out travelling, that’s another thing you have to think about cos you can’t go too far without needing the loo and it’s instantaneous so all those things. Personal things. They come into play. Even in your own personal life with your wife y’know, it a-a warm embrace with your wife or any intimacy you have to think about things with your hand shaking and it’s irritating and to sleep with somebody, and I have to put my hand under my leg under a cushion to squeeze it and it surrounds it. I’ll describe it with this pillow here. And it dampens, it dampens the tremor. I’m always very conscious my wife has to get to work. SO my sleep patterns are very broken so I get up and go into the little spare bed so I don’t disturb her. And then I can’t get to sleep again. So sometimes you feel irritable and anxious because you haven’t had enough sleep. So it’s all those little elements that come in together.

**CL- That’s really helpful <name> thank you.**

Alan **-** That’s alright.

**CL- I was wondering, same sort of question on a slightly different tangent, on a more personal level, can you describe how anxiety affects you?**

Alan **_** In terms of my physical?

**CL- It could be your physical, emotional, you thinking abilities.**

Alan **-** Erm I don’t feel as sexually engaged. There’s something that dampens that feeling. Because I felt depressed in the early stages. We had difficulty with that function, and that in itself gives you a kick and your thinking blimey and that makes you worried that you aren’t going to be able to deal with that again. So, it-it can affect your sexual function. I know it might be embarrassing for you to hear that.

**CL- Not at all.**

Alan **-** You can’t help it and then it leaves you feeling as a man, are these things going to be haunting you forever more. Then you can have performance anxiety because of the Parkinson’s and not sleeping right. Then you’ve got the business of how does your wife feel about that. You’ve got the problem of being able of being able to walk with her and hold her hand or be able to lay your hand across her body or something without the hand shaking. And, it-it’s really unpleasant and that causes you anxiety, definitely. Definitely. You just wish you could, you just wish you could go to sleep and wake up and you’re ok. That’s a good, you have this feeling of I’m fed up with this and it’s really getting on my nerves, I wish I could go to sleep and wake up and I was the person I was before all this happened.

**CL- Can you describe how anxiety makes you feel?**

Alan **-** It makes you feel, it can, it can be tiring because you’re always thinking. You’re always thinking about something. About Parkinson’s, how you’re going to deal with it and what it means to you. For me. And because you’re always trying to keep busy to keep your mind off it you can feel fatigued and your sleep, it uh. The sleep thing is a big issue as well. And also yeah, sleep is a big thing. It can make you nervous. It can make you feel nervous. More recently if I go into a public place to eat it’s becoming more difficult for me, I mean you can see if I’m trying to get peas onto my fork and you’re trying to sit in a restaurant and eat with your wife like you would’ve done. And, eh the knife will bang on the plate inadvertently because your hand is tremoring. SO that makes you feel like everybodies looking. Not that it worries me that their looking, its all to do with you perceive what that means for your wife and f’you you know. People do look and their wondering what’s going on. So you have to do things like put the blade between the prongs of the fork and flatten them so you can turn them and flatten your knife without making a noise. And stuff like this. Their funny things but you find yourself find ways to deal with it.

**CL- And those ways to deal with it, are they to make you feel less anxious or are they to reduce you, being less socially visible.**

Alan **-** I think it’s, partly, both things. You feel anxious that you’re making a bit of a spectacle of yourself although I don’t, for me I don’t care, what I’m trying to get at your mind is saying to you you need to try and quiten that down if you like. And-and your trying inadvertently not to be conspicuous because it’s not normal is it. It’s not normal to be different at the meal table we’ll say. Or it’s not normal to be when you go to pick up a glass of orange squash or so or beer and you and, yeah. So there are all those types of things that come into play that make you think and make you feel. Yeah, anxious. Anxiety is a funny thing because it can mean lots of little tiny things. It doesn’t have to be one anxiety is I’m biting my nails everyday and it’s just down to biting my nails everyday cos I’m thinking of a problem. It’s a mixture, do you know what I mean? It can culminate, sometimes you can feel really fed up at the end of a week and other times you can feel ok.

**CL- It sounds like a very complicated and complex picture with lots of different strings attached to it.**

Alan **-** Yeah you’d be right in saying that. It might be different for another person. If you…. I think it makes you more concerned having Parkinson’s. You dwell on things more than you did before. I hope that’s helpful.

**CL- That’s really helpful. You also mentioned sleeping a couple of times. In a couple of ways. You mentioned how being anxious can make you sleep less and that makes you feel more groggy.**

Alan **-** Yeah because your mind, I’ve had lets say for the sake of an evening if I went to sleep too early, and then say I dozed off on the couch after meal which after a busy day and you sit down, if I sit down and doze off I know if I sleep at that time, if I go to bed I try to go to bed between nine forty five and ten thirty. Religiously. I don’t like to sit up and watch TV or whatever, never have done. I try to stick to that because it takes me time to get to sleep and I have to be in the right mind to sleep. If I start thinking about anything related to the day or anything that might be stuck in my mind err about Parkinson’s, why the hell have I got Parkinson’s we’ll use that as an example. Why the hell have I got Parkinson’s, that will keep me awake. So you need to make sure you’re trying your level best, take the tremor out of it, to get yourself in a position where you’re ready to try to go to sleep. Then you have to try to find the most comfortable, comfortable position with the tremor to let you go to sleep. So you can drift off. And my wife has noticed that she can tell when I am asleep because the tremor is gone. There is no tremor.

**CL- So that is your signal that you’re asleep, the tremor’s gone.**

Alan **-** It’s gone completely.

**CL-Ok.**

Alan **-** So, I can have situations where I have dozed off because I feel and I’ve gone to bed at the right time and then all of a sudden I wake up at half past two thinking about something. And then because you’ve got to get to sleep again that can take you an hour, hour and a half. Even had to get up and make myself a drink and even make myself a sandwich to feel like I’ve had a meal and I want to go to sleep again. Erm. All those things make life just a little more tricky. I-I would love to have the feeling that I could go to bed at nine o clock and don’t wake up until seven o clock. I don’t, I don’t get up and generally like some chaps do to go for a pee. I-I sleep right through. Unless I’ve had a couple of beers or-or something like that then I might wake up at four or five o clock. But intermitten’ly the sleep thing is a big issue for people with Parkinson’s and their anxiety because the more their tired the more difficult it is to deal with things the next day, and the more your tired your not dealing with things because your tired then you get anxious because your not, you’ve not achieved anything. Do you see what I mean?

**CL- Yeah.**

Alan **-** It feels like that to me.

**CL- That’s how it feels to you.**

Alan **-** Yeah.

**CL- I was wondering if you could explain to me how do you react to anxiety?**

Alan **-** That’s a good question. Generally I think I’m ok. I can be short tempered. I can be short tempered. She can say well there’s no need to get you, too heated about it. That’s my wife by the way. She’ll say there’s no need to get so cross or concerned about something or make, I can fixate on an issue and it will make me feel irri’able because it’s not being dealt with. And that’s an anxiety in itself. I think that, I think part of the whole syndrome is because you’re not doing your usual things. You’ve been taken out of the norm of life, getting up, preparing yourself to get up, go to work, have a day, come home, prepare for your meal, wash, change, discuss things with your wife, go out, come back, whatever your doing. Your life is changed because, I know people who take three or four tablets three or four times a day each. And remembering all the medications and complications that come in. And making sure that you have exercise, and making sure that you have sleep. Making sure you allow yourself the time to rest because if you’re like me and you’ve always been energetic, and then suddenly you’re not doing your proper work your concerned your gonna turn into a lazy …. The-the one thing you do come to realise is that without rest it becomes more and more tiring. And that feeds the anxiety. Because you’re tired. And then you get anxious about things and then you think did I do that, no I can’t be bothered now. Then it exacerbates and then you’re always thinking then. Why haven’t I done that? Or I’m not going to do it? Some days I just say, to hell with it. I can’t be arsed.

**CL- Is there anything that you find is particularly helpful?**

Alan **-** I think that it is important to try and do the things that you like. Your hobby. Like mine is fishin’. And walking. And I think to-to make the effort to go and to do things like you would’ve done normally. And, even with a tremor I find it difficult to do things but I still try to do my hobby. I still try to find a bit of time to be away from home, which is very rare cos you can’t be off spending money and goin’ out and playing around when you’re not earning any money. Which I’m not at the moment. Erm. So. Yeah I think, that’s the best way I can describe it really I hope that helps.

**CL- That’s really clear, thank you. I really appreciate the time you’ve taken for this interview.**

Alan **-** That’s ok.

**CL- Is there anything else you think would be helpful for me to know?**

Alan **-** I think, if you were saying to another person, or other people with Parkinson’s what can we do to help this person emotionally and mentally. Is that a, they need to be able to discuss things with other people outside the home, and other sufferers. SO they, because they understand. And being able to go to someone and say you’re having a shit day heh heh, without anybody saying oh no not you again d’you know. A good group of friends and definitely to have exercise, and preferably to have exercise with people which we found, and that’s proven with the PD Warrior we’ve got one guy who lives out at erm Woodgreen Hale, <name>, he was unsure if they even wanted to come to the group for whatever reasons, their own personal reasons. But the difference in him coming to the group, you’re all open to each other. Everybody can see what each other is doing and how succeed and fail in certain things, and you live off you feed off of each other and give each other confidence. And that’s a big help. To know that there are other people with the same problem, getting by. And if you said to them I’m having a crap day they’d understand. Or mentally your not feeling good or, I mean my friend <name> he feels emotional and that’s all because of the Parkinson’s. He feels emotionally drained sometimes. We’ve had it where we’ve been to a BBQ and the musics been on, ad he’s had his back to me at the BBQ doing cooking. And he’s standing there and I’ll say you ok <name> how’s things. And I’ll turn around he’s got tears in his eyes, he’s heard the music and he’ll say I’m really glad I’ve got friends like yourselves that we can share and have these good times. It’s a bit worrying about what the future holds. I’ll say it’s only the Parkinson’s that’s making you feel like it. And we hep each other like that. So that’s something that might be useful.

**CL- Do you have any other questions for me?**

Alan **-** Can you please help the people find a cure for Parkinson’s hahaha. I think the biggest thing I’ve discovered since I’ve been diagnosed is the lack of funding for research and help for people. There’s a lot of help for all and cancers done extremely well and erm Parkinson’s is a, I don’t think we’ve touched the tip of the iceberg and now I’ve been diagnosed you see people with the same symptom and they may not even be aware you probably know you can have it for 10 years before you know you’ve even got it! So the research and the people you go and see their not clued in to what’s going on. I’ve asked professionals, I won’t say who because it’s unfair, but I’ve questions and they’ve said I don’t really know. Me and my friend we’ve both asked questions related to Parkinson’, things like related to this drug, where do we go from here and they’ve said I’m not really sure but we could find out. I’ve even known them to phone my pal up cos he’s been to a lot of these meetings for the, what do y’call it, stem cell research, the foetal cell one. They’ve phoned up and said <name>, from your latest visit to the seminar what’s the latest information on. Dadada. What I’m trying to get at is the people who should know your medication needs to be for you at your stage this, they can’t say that. So you feel anxious well if they don’t bloody well know, who does! I only cam on to taking my prescribed medication now because I saw a woman at a function who had tremor and she said her tremor was helped with sistravi.

**CL- Right ok.**

Alan **-** So, so I put it to my consultant, well we could try that. I’m thinking to myself, well we’ve been going along on the other tablet why haven’t you said try that. I, I don’t know. So, there are things like that that doesn’t instil a lot of confidence it’s a difficult disease to work with but I think when knowing full well there isn’t enough funding going in and there isn’t enough collaboration between all the consultants, sorry the researchers because obviously if they develop something they want to be the first to, to put it onto the market and I thank the lord that I have been offered up for this deep brain stimulation because I don’t know how I’d feel if I didn’t have that goal in mind. Particularly since my tremor has got worse. And that’s, that, that would make you extremely anxious it would me.

**CL- You’ve mentioned deep brain stimulation previously, does that make you feel more anxious? How does that make you feel?**

Alan **-** What? To go and have it done?

**CL- Yeah, what are your feelings around that?**

Alan **-** I feel very glad that they’ve developed something that’s proven to help people with tremor and other aspects of Parkinson’s as well. It can help just because your, you, your selected doesn’t mean you will end up a candidate. Because it relies on so many different factor. But if you’ve got tremor like I have and you can see my hand. Shaking like crazy here and you put up with that every day, day in day out. I’m very excited at the prospect of going and being treated and something will hopefully happen. There’s a 90% chance it will do me good but the risks, the risks are there. There’s a high risk of-of by it’s very nature putting a probe through your brain to a point down deep in the brain, that you hit a blood vessel and give yourself a stroke. Or- or what do you call it a neurological problem. Or putting it in slightly the wrong place and turning it on and it can cause your eye to drop or your speech to be wrong or something else. So yes that makes you anxious. You can’t odds that feeling but you have to weigh up or try and get some consensus as to you know am I prepared to do this. You here is a situation for you the news has bought to us that there’s a non-invasive system where they fire electrical impulses into the brain from several points into a point into the brain and it stimulates or destroys an area where the signal is getting changed. So for a normal person your brain your mind is telling you to be still like you are now. My brain is telling most of my body to be still apart from my right arm. So without having to go into my brain which they possibly can do that which they are saying they can do for tremor. Essential tremor. They may be able to help people with Parkinson’s, I’ve got the thought of well, have I, if they’re developing this do I really want to go in and have something pushed into my brain. So that in itself can make you feel anxious. It’s a worry. You have to try to make an informed decision. And it’s a big decision. It can affect your life instantly. It can either be for the good, or not quite so good. So, that on top of actually having the disease is a worry.

**CL- Hmm, quite, absolutely.**

Alan **-** So.. but when you level it all out you have to go with the professionals and their knowledge and their expertise and trust them. And you can, you can actualy get run if if you run out of your door.

**CL- It’s weighing it up.**

Alan **-** So, how how do you deal with it. And that’s really about it in a nutshell really.

**CL- That’s really helpful thank you <name>.**

Alan **-** That’s alright.

**CL- Is there anything else you think would be helpful for me to know before we wrap up?**

Alan **-** Erm. I don’t think so. Just to keep an open mind and to keep trying, try to keep positive. Try to.

**CL- Would you like a summary of the findings?**

Alan **-** Yes I would like to know what’s gone on.

**CL-Thank you. I’ve got all of the information that I need. I will now stop recording. Thank you again for your time.**

Alan **-** That’s alright you’re welcome. Very welcome.

<recording stops>
