# Supplementary material for: What is the lived experience of anxiety for people with Parkinson’s? A phenomenological study
Source: PLoS One. 2021 Apr 8;16(4):e0249390. doi: 10.1371/journal.pone.0249390 (PMC8031398; doi:10.1371/journal.pone.0249390)
Supplement: S3 File — (DOCX) [file pone.0249390.s003.docx]

**CL- My name is Chris Lovegrove. I'm doing this research with Plymouth University. I would like to ask you some questions about your background, your condition, some experiences you have had, and about you. You do not have to take part if you do not want to. I hope to use this information to help to develop more anxiety interventions specific to people with Parkinson's. The interview should take about one hour but may be a little shorter or longer. Please feel free to ask any breaks that you might need during the interview. Are you happy to continue?**

Beth- Yes.

**CL- Let me begin by asking you some questions about where you live and your family and yourself. Are you still happy to go ahead?**

Beth- Yes.

**CL- So, can you tell me a little bit about yourself?**

Beth- Well I’m a retired teacher. I’ve been retired since 1997. I was eh diagnosed with Parkinson's in 1999 but it wasn't a surprise to me at all because I told the doctor that I got it he didn't tell me. I said I’ve got it and he said “no you haven’t you’re the last person I would think having Parkinson’s and I don’t want to be the one to tell you you’ve got it”. But I persisted and went back to him after two months and “okay I'll and refer you to a specialist” and he said yes I think you have, got Parkinson’s. Having done, you know, all the usual hands-on tests. And later on much later on I went for another diagnosis in 19- no, in 2011. I wasn't happy with things and I went for another diagnosis. He, he said to me “no I don’t think you’ve got Parkinson’s, you’re the last person I think would have Parkinson’s!” I thought, don't be silly I know I’ve got Parkinson’s I know I've got it. And then I had a brain scan and of course it was confirmed that I had Parkinson’s heh heh.

**CL- Ok, so you mentioned about you you told the doctor that you had Parkinson’s.**

Beth- Yes.

**CL- What things we were you experiencing that made you think that?**

Beth- Stress but I-I I always knew I was going to get something like that. I don't know, don’t ask me ask me why. I I was an adopted child and there was nothing in the family to do with Parkinson’s but I always thought that one day I would like to to contact my side of the family. So when I decided, I had such a lovely mum and dad I never bothered to do it. But when my daughter was going abroad, my-my mum and dad by then, they’d gone, I decided that I’d occupy myself in some way. So I looked for my family and to my, not to my surprise actually found my father, my sister told me, cos I had a family, I had three… I'm shaking at the moment because I’m talking to you that’s why, anxiety does that. I’ll calm down in a minute I’m not usually as bad as this. I had a family, mother and father three sisters and brother, which I found in the beginnings of the 90’s, just about the time and I was becoming aware that I had Parkinson. But then he should tell me my sister tell me our father had Parkinson he had young onset Parkinson's. He died in his 50s in the time no one at that time there was nothing. He must’ve had an awful time. He went to America to stay with a brother of his who was a dentist. I think in America that time they were doing frontal lobe operations and he went through the idea of having this operation apparently then he understood what it was all about and decided not to. He came back. That that's the sum of it. It’s an inherited thing and somehow I always thought I would be done i-i-if I was in a doctors surgery and I saw something about Parkinson’s on the wall, a cold hand used to grip my heart. It’s still so strange but it was almost, I knew I would develop it. I thought I would. Does that make sense, it sounds a bit daft. But that’s part of my my experience.

**CL- I'm going to ask you to tell me a little bit about your diagnosis with Parkinson's and how you felt about that. You mentioned about how you weren’t happy with the things that the doctors were telling you. You went to some other consultants. Can you tell me about that process and how it made you feel?**

Beth- I can’t remember exactly why I wasn't happy. I I wasn’t happy with the treatment that we were provided with. When I was diagnosed with Parkinson's start with we had a very good doctor he was very good and I saw them on a regular basis there was a Parkinson’s nurse I saw regularly. And I felt I was being attended too and helped you know, although in it is a lot of things that they can’t help you with. I was quite happy with Parkinson’s but I didn't, I had a wonderful drug called ropinerole which I had asked for because I'd read about it, you know, in the papers. And I asked for that drug and he said yes you can have it. It agreed very well with me. I didn't take any drugs for two years. He said you can have drugs I said no I don't want it. I want to go as far as I can without them. And then the two years after diagnosis that I decided to take ropinerole which I was on for several years, which was good and eventually I was on the top dose 24 mg. I had to give it up because my it was beginning to have side-effects my legs were beginning to swell, so I was I was soon taken off that and then I became dissatisfied and I can’t remember exactly why I went for another diagnosis. I think I wasn't happy with the drugs I being given and but I was most surprised when he told me I was the last person that Parkinson. But I did used to fall. I was always always racing around Bath because I love looking at the shops but at times I would fall flat on my face. Right in the middle of Bath. I’d find myself on a chair with everybody around me, ooh we must ambulances this that and the other no no just get away from me, go away. Please I just want to go home. It manifested itself as these pains in my legs and they're not shaking so much I don't shake a lot I am at the moment this is because I'm talking to you. But that’s not, it’s stiffness really. And my foot is turned out completely. Walking is, balance, balance and stiffness is the, are the other main difficulties I encounter. Last year I was in hospital for, my walking my falling got so bad I was falling about 10 times a day! I was falling a lot and I had this <points to pendant alarm>, in the end I was calling them almost every day it was getting ridiculous. And I would knock my head against the fridge. My head was bleeding a little it was nothing, but they insisted I went to hospital. I went to the hospital and they got the, there was a slight cut in my head. That wasn’t attended to at all they were more concerned about my balance. And when physio saw me and said we think think you ought to be in hospital for a week or two to stabilise you, you’re stumbling every every step almost, so I agreed to do that. Then something very very strange happened, I became delirious. And I was delirious for six weeks, coming in and out of consciousness. And I had pneumonia and I had a stomach bleed all out of the blue. And I was, my family were told three times that I was gonna die.

**CL- That sounds like a really traumatic journey.**

Beth- It was terrible actually. Three months in hospital they put me on a side ward, wouldn't take any notice of me, then just left me. I remember that night, awful nightmares I don’t want to go into it, it is not what you want hear, but I had these terrible nightmares. Terrible terrible time I had for three months and began to pull through after about six weeks. I turned the corner and they said it was a miracle. I was determined to survive. All the time I was fighting. In fact I became aggressive and I bit people. Heh heh it sounds horrible but then personally following but I was like an animal, I regressed. I went went back to being an animal to almost my basic instincts. I wanted to escape them they were forcing me down on the bed and they were making me have things on that I didn't want clothes on that were hot. All these people I just l wanted them to go away and leave me alone so to protect myself I bit people. That was instinctive. It was sat- I had gone back to the basics really. Anyway I pulled through. Then I had three months in a nursing home which was far worse than the hospital. Nursing homes are deadly places. And after that I thought if I had stayed I would’ve died to be honest with you. They’re awful places. You’re just on your own in a room and your alone

saw what happened to some other people. It was quite horrible and and they let me, the social worker she fixed me up with a wonderful care package. I was told that I could come home, because my husband can’t look after me, that I could come home and have care twice a day but what she did fix me up with was a night care, all night care. And that was absolutely fab that got me through. I was told I had to come home sit still until the carer came in. Well I couldn’t sit still. I was told if I went to the toilet I had just had to go to the toilet. That’s not me I’ve just gotta, I had to get up and do it. I survived because every time I've sink down, I’ve felt down at the bottom sometimes, everytime I thought what's the alternative nursing home no way! You know the only way is up. So I've managed three year now and I'm doing quite well. In fact I’ve just done something very very risky and I’ve been doing this morning.

**CL- What’s that?**

Beth- I’ve arranged to go on a cruise. I’ve got such a wonderful carer we went on holiday for six days in Torquay and she is so marvelous. She’s a Romanian lady, doesn't speak much English but I’m encouraging her to, you know, to get on with her English. She’s a marvelous person she’s full of life and joy and happiness and she's a a survivor of lupus. This is she's pulled through that you know. Anyway I have been arranging that this morning. I must be completely mad but I think what the hell.

**CL- You mentioned your carer being full of joy and happiness. Would you say those qualities are important in others to help you cope and get by-**

Beth- Very important, very important. Love of life write and and a will to, to get on and do your best and she's all that. She's encouraged me a lot then she got down a bit the other day and I was like go on and get out and go for a run or something. So we turn the tables through few days is so she was beginning to wonder she had this Lupus coming back by. But she she had a very bad cold. I gave it to everybody very generously. It got her down a bit but she’s ok again now. She’s fine. Sorry I’m going on a bit I can talk and talk so shut me up. **CL-Could you please tell me a little bit about your typical day?**

Beth- Typical day is not very exciting I’m afraid.

**CL- That's ok.**

Beth- my typical day is existing really, getting getting by, getting, doing things with success. When anxiety comes in you, first of all I must tell you that I've always been an anxious person. And, anxious about things my son thinks that I get very anxious about things. I think he overdoes it a bit sometimes. I have I used to when I was doing my A-levels I have suffered a bout of anxiety there was nothing for it in those days, so strongly I went to bed and decided to die. It was high levels of anxiety which overcame me completely. My heart used to beat so fiercely I thought I was goin to die. So I’d about I become anorexic from various reasons, all sorts of difficulties there, and I became highly anxious. I was, I was wanting to escape my A-levels to be honest with you. I didn't want the challenge but I didn’t die after a fortnight so I thought I better get up and get on with life heh! So I did. But a couple of times when it came to teaching practice the same thing happened to me and I went to the doctor, I was dying ooh you need a break my dear.I’m not going to give you a break he said I’ll give you a chemical break. To get you into the classrooms. I did my teaching practice late and it’s one of the most terrifying things a student has to do, to go in and face a class of kids. Because they could make mincemeat of you! And some of the things you had to do, the schools weren’t very good, they were appalling. I took the pills throughout the week ago and they, they got me into the classroom. This is what I tell my grandson because he's got trouble with anxiety, and it got me into the classroom and I thought, don’t need the pills, stay in the draw you know. I'm trying to say that I had anxiety before the Parkinson’s. I just wonder if the anxiety though, and the Parkinson's were actually there from the beginning. I often think that Parkinson's perhaps with you before you know about it long, when I was a child even I think perhaps it was there.

**CL- So now you know your diagnosis of Parkinson's, what is your experience anxiety with that?**

Beth- My biggest anxiety with Parkinson’s is that I'm not be able to cope , my big thing daily. My daily thing is balance. And there are times when my balance is poor. And I have to balance I have to get up and walk and I have to do it in the way, I have to get there but it but the anxiety is there, high level of anxiety. But deep down I know I can do it. I have I tell myself that I can do it. I think it out so that when I was in-in the nursing home, I had to think out my every move because nobody was helping every time that I asked for help. Sorry dear I have someone else to see too. Somebody dying or somebody doing this or other, you never got any help. So I used to plan out my moves. How am I going to undress myself. I’d plan out my moves. Anxiety is useful sometimes as it makes you plan.

**CL- Ok.**

Beth- It prepares you the difficulties. You don't rush you sit there and plan it out. And walking I have to think, sometimes when my balance is very poor I have to think what what's the best next step I can make. Am I going to be safe? Because there’s an awful lot that depends on me being safe. If I’m not safe and I fall, I don't want to have to go back to hospital. So it's really important that I keep my balance and that's an anxiety. I have to forget it sometimes. I forget things. A wonderful thing helping me at the moment is Tai Chi. It’s helping my balance considerably. I've sent away for this tape and at the beginning of it, I have got, I’ve got frames on my bed. I was clinging thinking I can’t do this I can’t let go of this. Then I can do, on a good day I can do the whole thing without catching anything hold of anything and I can move from one foot to the other which is absolutely miraculous. In fact I was so impressed with it that I’ve written and told them how well it seems. I would recommend that for anyone. This particular one, this particular one is very helpful and it is designed for people not just the elderly, they mentioned Parkinson and it works. So that is a great deal helps helps a great deal with anxiety. I've got a iPad Pro which you know, take me to all sorts of places, and music helps a lot.

**CL- How does music help you?**

Beth- I got up and dance to it at times heheh. In the nursing home I used to dance in my wheelchair. And I tell you what I’ve had speech therapy cos my voice isn’t good and and I I hope it will come off, I’ve had practising you know which helps getting my voice, and and so much stronger . I used to have a very good voice as a teacher which is important and from going from a very strong voice. It sounds horrible going mmmmmmmm. It sounds miserable. So I've done practice with it and singing, I want to join the choir. I haven’t worked out yet quite which choir and how I'm going to get there. This is the big thing with Parkinson's. Like me you can’t go out on my own. They said to me before she’ll never walk again she’s been too long, you know blinds on the back, but I was determined that I would. And so I do and I'm not a good walker and as I say my balance is a big I can’t go out on my own. I have to have somebody with me I get out and I get into the car. I can do that. But I couldn't walk round the supermarket with my walking frame I can walk around the house and walk to the car but I wouldn't try beyond that. It’d be foolish. You can do things that are daring but too daring would be stupid it would put me in jeopardy and other people as well. So you know there is a limit.

**CL- Do you find you take less risks because of anxiety or do less daring things, or is that purely to keep yourself safe?**

Beth- Anxiety in a way helps because I don't do silly things. I mean I was doing silly things before. I was taking risks. And anxiety of keeping myself intact is important, it makes me be careful. I've got to think about the next move. I sometimes get in position and think, now they make me have this on <points to pendant alarm> that I don't want to call them whereas before I was falling stupidly all over the place and being silly about things, now my anxiety is useful. It’s not pleasant. It’s very unpleasant but it's useful. Does that make sense do you understand?

**CL- Thank you that makes sense it’s very clear. You mentioned some things like music, using the computer, things like that. Do you find those things helpful for managing anxiety?**

Beth- Occupying your mind is important. I'm lucky as I suppose I’ve got quite good memories. Not a bad mind really.

You know I’m not sort of forgetting things. The one thing I don't do as much of which I must really try to get back to is reading. I was an avid reader. My iPad has taken that over from me. And I’m afraid I spentd too much money I’m always on the Internet buying something, which is, my consultant always asking me are you spending too much money? I say well no I've always been spender hehe. But I’ve got too much time now to spend and now this proves that I've really got to I've got to change my mind set and really save up. The booking a cruise has given me something to look forward to. At my age you know I haven’t got an ambition. Or my ambitions have to be very minor if you know what I mean. I have a mind that needs to think about something, to look forward to. The thing I miss more than anything is social-socialising, but I have a lot of socialising actually with my carers. I'm very lucky like that. I think the most deadly thing for anybody is nursing home.

**CL- Why do you say that?**

Beth- It is terrible! It’s a terrible place! In hospital at least you get to see people, there you’re just in a room and your isolated and then the, the food is dreadful. Because care has gone the wrong way it’s not run for the people it’s run as a business. The poor carers there have no money at all, hardly anything, you feel sorry. At night there were two carers for 28 residents on one floor. Nobodies got time for you. The old lady next, across the way with, 93 and she was on her feet and she was, she was a little forgetful and things but not terribly. And she was at the door saying please can I walk along the corridor, please can you take me just down the corridor. All they wanted to do was say sit down dear, sit down. For their sake not for hers. Nursing homes are not humane really. And I would suggest that anyone if they can should be at home. They've got everything they need there basically. Home is where you want to be. And for a person with Parkinson’s it must be sheer hell to be in. I know there are people worse than I am. I’ve had Parkinson’s now for years and I know I’m quite lucky in a sense. I know there are people much worse than me but oh they are deadly places.

**CL- How does anxiety affect you?**

Beth- Well mostly as I say with my balance. I don’t allow it to affect me too much because if I did I would be overcome. So I've got to find, get, in fact because I'm so slow about everything, you know I’ve got to be careful about walking to the bedroom and getting there. And dressing and washing and everything you do. Slowed up. So my day is taken with very often with quite mundane ways if you what I mean. I help with washing and dressing. Going to the toilet, cleaning my teeth all this takes me much longer than normal people would take. There’s anxiety there to a certain extent, am I going to do this properly. And sometimes the anxiety is really there when you’re out-of-control and you’ve not got control of your body. There are times may condition fluctuate wildly, there are times I’m rushing around there are times when I feel I haven’t got got control and that's that that is very anxious making. That's where you try, to try get yourself up really. Do something, look up something, find out something found out, phone somebody, find an occupation for yourself. I think people matter a lot. And I would like to socialise more than I do. If I didn't have all those people there to help me and I was just in a nursing home stuck away, I don’t think I’d live that long to be honest with you. I think people are important, well they’re important to me. Because I like talking, I like listening and talking to others. I'm able to do that. My night carers, one of them is a very interesting South American lady and I like listening to her talk about her family and her life in Venezuelan and all this. I have all these extra interests because of my carers. You know. I make friends with my carers. My daughter wasn’t the keenest she said they’re carers you mustn't you can't be too friendly with them but there has to be I suppose some. But you can’t help being friendly with people can you! How can you stop yourself and say this is a carer I have to behave. I have a carer of about 23. She’s great. You know. I could not, most carers of good and-and you treat them as your friends. And that, the carers help a lot.

**CL- Ok. Can you describe how anxiety makes you feel**?

Beth- Frightened! I was very anxious about your coming today. I was thinking, I don't think that I can remember we told me I had to read. Then I thought what does it really matter and I was, I woke up very early because I had to get all my papers together. I was also trying to work out this cruise thing which has also given me a lot more anxiety. Undoubtedly I could be sat having a peaceful life not doing anything but I've always, I make myself anxious sometimes. I do anxious making things. Because they’re interesting, they give you life. If you have nothing to worry about what you, you could just sit there like a zombie couldn’t you.

**CL- How you react to anxiety? When you feel anxious how you react to it.**

Beth- I think that the next, well, my anxiety is generated as I a say through movement. And I just, I just get that make sure that I’m safe, to make sure that I'm not without some kind of support when if I need it…

**CL- When you say support, what would that look like?**

Beth- Well I’m meaning physical support really. Sometimes I find myself, if I am really active and I’m on a roll I find myself out in the corridor without the walker. My social workers who I rarely see said <name> you’re letting go of your walker! Then one of my night carers first of all said come on <name> you can do without your walker. So I’ve got the the two opposites there and neither of them are right because the social worker is making me nervous and you could get you know you mustn’t let go of that, and that the other is saying come on, you can walk without your walker. So I've got to go between the two of them and say one’s too much one’s too little. In the end I’ve got to be answerable for myself. Other people cannot be responsible for me. And I think you’ve got to have trust in yourself. Faith in yourself. In the long run it comes to you. When I was came home first of all. My husband was used to spending six months on his own and he didn’t want me to ask him anything. If I dared ask anything he’d say oh go back to the nursing home. In a way having a husband that was not able to help, helped me, because I had to help myself. I think if you’ve got somebody running after you saying yes I’ll do this for you can I do that for you. You just let them. I think in a way I’ve got to be grateful for someone who doesn’t do much and can’t do much for me. Because you could become very, you could just sit there and say get this get that do the other thing, you could come to rely on somebody. It's very easy to say could you get that for me instead of making the effort yourself. I think having to make that effort is important. People shouldn't have things done to them. That is way nursing homes it is so awful when they keep people sat down. They are sitting down not for their own sakes it’s to take the weight off because they don’t want to be bothered with them. And I'm not saying they cannot help it, the situation is a bad situation, people are running nursing homes because they wanna make money out of peoples illnesses and disabilities. It's a world that’s wrong, if you know what I mean.

**CL- I really appreciate the time you took this interview. Is there anything else you think would be helpful for me to know?**

Beth- I think the important things are having to do things for yourself. But at the same time having support. They always say if you have Parkinson's never be alone. That's a load of rubbish! To be honest with you I don't find any of the organisations any help at all. I went to one Parkinson's meeting and, with my son-in-law, my daughter is always abroad. She’s queens messenger. You heard of that?

**CL-I’ve heard the term, yeah.**

Beth- She’s only the second woman ever to have done the job. She's 55 and at the moment she’s in Thailand. Just the last three weeks she's been doing it. She's travelling she's very happy but how did I get onto that…

**CL- The organisations you don't find very helpful-**

Beth- I tell you another thing with Parkinson's, if you get emotional upset. I had an emotional upset, my son-in-law is very difficult person and he upset me very much one day. He does it purposely he goads me and in fact there was so taken aback so upset that I couldn't walk. It effected me in that way and I was sitting there and I couldn't get-get myself up. I think I-I asked the doctor to come and see me. I forget things because they they, I forget things because I-I wonder, that's finished and I’m going onto the next thing if that makes sense. I know the district nurse came down and she said, she said you're not safe. She said you’re just sitting there and she said you're not safe and I think you ought to be in hospital. What, I said! No way am I going in hospital. The doctor came to see me and I said no way am I going back to hospital. And I thought just to pull myself together. The consultant works in a way unlike the other one, he, you have to go to him rather than him giving you an appointment. Which I wasn't used to. I was used to knowing I got an appointment, this one works a different way. You ask for help. I didn't understand that and nobody explained it to me. I thought it was neglect. But he said there’s no more medication at the moment. It was a case of get on with it. Then you've got to get on with it, I think, it's important… I’ve lost what I’m saying…

**CL- You were talking about being upset and getting on with it.**

Beth- Yes, being upset emotionally can really affect you. And it can bring you right down. So it's important you, you have someone to talk that through with. And that's where carers come in to a certain extent. It depends on the carers of course you couldn’t talk to everyone. If it's a good carer and you can, your, they can be much different to you they can be much younger, much older, they could be the same age the same age. It doesn't matter what age they are that’s of no importance to it’s the sort of person they are. There are some carers you wouldn’t say a lot too, there just not the same. I think carers in Parkinson make a lot of difference, that support is I think it essential.

**CL- You mentioned a situation of your son-in-law that was quite upsetting. Is that sort of you receive support to manage?**

Beth- I don’t think I do really. It was just a sort of clash of personalities. It probably is that to a certain extent but he’s not an easy person to get on with. My daughters always abroad.

**CL- Do you feel at risk at all in that relationship?**

Beth- Not really, only that heneither he gets at me you know. In fact I hit him once, and he thumped me back. Not very hard I didn’t take issue with it. But now I’m very careful what I say to social workers as they take every thing up immediately.

**CL- That situation, did they manage that at all? Where there was a physical altercation?**

Beth- I mentioned it and the social worker took it up and said it’s got to be bought up at a meeting.

**CL- So has that particular issue been resolved now?**

Beth- Well it’s resolved in the fact we had a meeting and I don’t spend too much time with him heheh.

**CL- Ok. I need to double check with people as a healthcare professional that that isn’t a new thing I have to take forward.**

Beth- Yes, no no it’s all alright now.

**CL- Do you have any other questions from me?**

Beth- How useful has this been to you?

**CL- It's been really useful. Really really useful and again I’d like to thank you very much the time you’ve take to talk to me. Would you like a summary of the findings of this study?**

Beth- Yes I would, very much so.

**CL- I have all the information I need. I will now stop the recording. Thank you very much again.** <recording stops>
