# Supplementary material for: What is the lived experience of anxiety for people with Parkinson’s? A phenomenological study
Source: PLoS One. 2021 Apr 8;16(4):e0249390. doi: 10.1371/journal.pone.0249390 (PMC8031398; doi:10.1371/journal.pone.0249390)
Supplement: S4 File — (DOCX) [file pone.0249390.s004.docx]

**CL- My name is Chris Lovegrove and I'm doing this research with Plymouth University. I would like to ask you some questions about your background, your condition, some experiences you've had and about you. You do not have to take part if you do not want to. I hope to use this information to develop more anxiety interventions specific to people with Parkinson's. The interview should take about one hour but it may be little shorter or longer. Please feel free to ask for any breaks that you might need during the interview. Are you happy to continue?**

Clare**-** Of course.

**CL- Let me begin by asking some questions about where you live, what you get up to and your family. Are you still happy to proceed?**

Clare**-** Yes.

**CL- Ok thank you. So can you tell me a bit about yourself?**

Clare**-** Well I'm 61. I’ve got three daughters. Two of them live near here and the other is only in North Devon. So I’m very lucky and the two that live here have got children. So I’m on hand for the grandchildren. I play tennis, not as much as I used to but I still play. I used to be a primary teacher, but I stopped, I stopped working four years ago mainly because of Parkinson's. Um not so much the Parkinson's itself but stress and anxiety. Um I was just I wasn't coping in the classroom so I was off sick for six months and then I took early retirement, which has been the best thing I've done heheheh. So I don't regret that at all. Um, I don’t know really. I live here on my own, my husband died 18 years ago. Um but I’m lucky I’ve I got some good friends and also my daughters are very close and I see them regularly. They’re are very supportive, and that's about it that sums me up really. Haha!

**CL- Ok. Can you please tell me a little bit about your diagnosis with Parkinson's?**

Clare**-** Yes of course. Um, I think it was probably about five or six years ago. I went to the doctors. I realised as I was walking along, as you can see we live on a hill, and I was walking in my left arm wasn't swinging. I suddenly realized I was making it swing because it looked weird, and also I was walking around at work carrying my left arm as if it was hurt, as if I had injured it. I couldn’t really understand why because I hadn’t hurt it and why I was holding it for. Anyway eventually after, I think I saw the doctor once or twice, and he referred me to <hospital> to the consultant. Um, and by which time I put two and two together and I'd read a couple of articles in the paper about people with Parkinson's and I had a bit of a feeling. I was finding it difficult getting money out of my purse, my writing was getting smaller, and it's never been good but it’s got a lot worse. And he actually said to me have you any idea what might have and I said I think I may have Parkinson's and he said yes we agree with you. And he put me on onto medication straightaway. He was very, very prompt, very good and I've been seeing him or somebody else every year since. So I think… I think I was actually firstly diagnosed in 2012, and I finish work in 2013. The September, September 2013. So I kept going for a year and a bit but I realised I just couldn't deal with it. Um I didn't used to have a tremor. It’s not so bad now but I definitely do have a tremor and it’s becoming more and more pronounced. It's a bit, bit annoying, and I do find I’m having a little bit more difficulty playing tennis. I can't serve cos it’s my left arm. I can’t serve, not that I was ever brilliant, but I can't service consistently as I used to. So that's a bit frustrating because tennis is my, my thing and I’ll be cross if I have to give it up. So I’m fighting that at the moment, yes. Um, but I do find there's a lot of little things like and doing buttons and undoing jars and putting shoes and socks on, and just silly little day-to-day things. I can do them but their increasingly fiddling and awkward. And, I went, just before Christmas I went to Australia with my brother to see a half-sister and I felt really useless of course on the plane all the food it comes along in packages, plastic packages. And, bless him, my my younger brother was having to undo all my knives and forks and things and all the little parcels as we were sitting there with our little plastic cutlery. He had to and it’s just silly stupid little things like that. It is frustrating but you just suddenly realize that you do need another pair of hands sometimes and it's just, just irritating. That’s, that's about it really. But apart from the anxiety which is, I never have been ann anxious person. I’ve have never suffered. When I was a primary school teacher you can't really be anxious and be a primary school teacher. It doesn't, it doesn't fit in with being a primary teacher and I’ve always been confident and outgoing and a people person. But um, it's before I was diagnosed I was standing in the classroom a few mornings, you know standing waiting for the children and the parents come in and gossip and chat away and I just suddenly realised I was standing there not knowing what to do with myself. Whereas I’d done it for 20 years and it never, it was just always natural, I was just feeling like a spare part in my own classroom. As I say this was just before I was diagnosed and it was, and it was just odd.

**CL- Just so that I can be clear that I understand, something you were normally very familiar with having done it for 20 years, you suddenly felt like a spare part?**

Clare**-** Yes. SO, um, with hindsight I-I well I believe that it is it is to do with Parkinson's and I was actually put on antidepressants before I was diagnosed with Parkinson's. So I think probably three or four years before, before my diagnosis I was put on a mild antidepressant just because of my mild anxiety. And I've never been depressed, but just from talking to people since, and reading up on it and talking to the consultant, you know it seems that the anxiety is very common. It’s a very common part of Parkinson's. I found that, social situations, and as I say I’ve always been a sociable outgoing person but, um, I think twice now before I accept an invitation. Or going out to dinner with friends I feel more, more uncertain and insecure than I ever have done and I still go usually, but I'm looking for an excuse quite often. Does that sound….?

**CL- That’s very clear thank you. I was wondering if you could tell me a little bit about when you went to see the consultant and you got your diagnosis, I was wondering if you could tell me what that experience was like? How you felt?** Clare**-** Yes it was it was quite weird because even though I had it in my mind that I might have Parkinson’s I wasn't really expecting to come away knowing, heheh, or being told I’ve definitely got it. It was, it was very strange, there was another, I spoke to the consultant first and then he left me with another lady his, what do they call them, his minion, you know his subordinate. And she did some few tests with me, my reactions you know my hands and legs and things, and she was there, both very sweet very charming. And then they disappeared together and obviously had their little conflab. Then they came back together and spoke to me and said that it did seem like my gut feeling was probably right and it was.. I can remember it was really weird because it must’ve been about 9:30 10 o'clock in the morning and I went back to school because I just had the first thing in the morning appointment. I went straight back to playground duty and I was there on my own with my cup of coffee, thinking, oh, shit! And there’s all these hundreds of children running around and I thought, oh, darn it. What have I just been told?! But it was really weird as it was a bit like it was happening to somebody else. It was a bit surreal. I don’t know if it was partly because of my antidepressants or anything, but I’ve never got emotional about it. I’ve never let it overcome me. But I do remember very distinctly standing in that playground, thinking I’ve just been given a bombshell, what am I going to do. And I did speak to one of my friends who worked at school. I did see her later that day and I just told her and got a hug. Then I came home and told the girls, and they, unbeknown to me they had a secret meeting about it hahaha because they’re good at that hahaha**!** They had an emergency family on my behalf, but I just thought you’ve got to get on, you know. I didn't realise I’d be giving up my job but I just thought you’ve got to get on with it as much as you can, as well as you can. And try not to let it rule your life. It's just one of those things and I do remember quite plainly one of my best friends was actually had just died from pancreatic cancer. It was horrible but I think what really kept me going and kept me feeling positive, you know I’m not going to die from Parkinson's. Which was my first question to the consultant and he said no you die with it, which was very reassuring! But I just thought, my poor old mate, he had six months and went downhill drastically and had the horrible death and I thought I’m not going to have a death like that. And I thought I’ve just got to be grateful that it could have been a hell of a lot worse. thank **CL- You’ve mentioned a few times about work after you were diagnosed. I was wondering if you could expain that a little bit more, and how that was, working with Parkinson’s?**

Clare**-** Well it was quite interesting because

I didn't officially tell people at work for quite a while. Then I thought maybe I should tell my headteacher. And then I think a month or two later, and, we, I told various people I mean I didn’t want it to be a secret. I don’t think that that’s a good idea. But it was interesting because there were no concessions made. I didn’t expect special treatment but… I don’t know if the school is the same as the health service but I don’t know. But your just to muddle along on your own. One thing I did do, cos I was PE coordinator, and and I had to, I used to organise sports day that sort of thing, but I thought after a few months that’s ridiculous having somebody who is not really very coordinated hahahaha. Being PE coordinator. So I opted to give up that role, well I volunteered to give it up because I thought it was a bit silly. Um but otherwise I just got on and wanted to prove that I could do everything the same as I always had done. And it wasn't until, it was the February half term, I'd just been struggling with the workload and just the pressure generally, and I had been coping, I got to the February half term and began thinking; I can't I just can't go back. And, and as I was I was awake at nights and thinking all sorts of negative things about school, and we got a new headteacher and we had a bad OFSTED and it was all. It was all really, really negative and stressful and I just thought no I can’t do this anymore. So I saw the doctor who was brilliant and he signed me off and I think I I kept seeing him every month or so. But then I during that time in the next six months I was in contact with my union, and a really nice girl took me through the, we decided things just before the summer that I wasn't going to be able to come back and she helped me to get early retirement through ill-health. So I did the whatchamacallit, occupational health. My daughter and my consultant backed me up obviously. So I just, I managed to access my pension early because otherwise I wouldn't be able to do it I had assumed I had to stay until I was 65, which would have been horrendous! Luckily I was accepted and, also I got, I’d had insurance. Life insurance with critical health. So I got a payout from that which enabled me to clear my mortgage. So with my pension it meant that I was able to live because otherwise I wouldn’t have been to survive. In fact it’s worked out quite well and I'm actually more comfortable than I have been for a long long time. So that that was a big weight off my mind and I think that helped just to know I had the financial security.

**CL- That process of taking early retirement, how did that make you feel?** Clare**-** Well in a way it was liberating. I'd been the sole breadwinner for quite a long time when my husband was alive. And we'd always been totally, totally skint all through my married life. And know that I didn't have to work in order to make ends meet was was actually quite, is quite nice but at the same time, and I was surprised when I had stopped working, that I missed the sense of purpose. I still do miss having something to get up for. Although I’ve got bits and pieces that I do, plenty to keep me busy but I still miss having that, goal because I'd worked while 25 years. I was never previously off sick I was the sort of person who was never ill and worked most of the year without any days off sick. So it was a bit of a shock.

**CL- Could you please tell me about your typical day?**

Clare**-** Now?

**CL- Yes please.**

Clare**-** Yes, um, well I wake up quite early. I think that's a result of teaching for donkeys years and being up at six or half six, so I usually get up around sevenish. I come downstairs and listen to the radio and get my breakfast and mooch around. Generally I don't lie in bed all day, I’m up and about. It’s nice because I don’t have to race and do anything. It depends what day it is but my two daughters who live here, I often see them during the week when they're off work. One of them is a nurse and she doesn’t work Wednesdays so I usually see her on Wednesday. And the other one I sometimes look after her little girl on a Thursday when she goes swimming. So I’ve got little bits and pieces like that that I do. And I go down the road every morning to get my newspaper which is the highlight of my day ha. I do Pilates on a Friday morning which I think helps my balance and core strength and that’s been recommended by a few people, so I try to keep, try to do that. I do my housework, grudgingly haha! I'm not the worlds best house wife. I well, I say I play tennis I haven’t played since I went abroad in November. In the summer I try to play once or twice a week if I can. We usually play on a Sunday morning. So I look forward to that but it is a bit more of an effort in the winter so I’ve been using the weather as an excuse. But I have got to get back into that. I don't know that there’s much else that happens in my typical daily really. I've got a couple of friends I walk the dogs with, I go out for walks with them. I might go over a girlfriends for lunch or coffee. It’s only a small circle of friends but they’re good friends. Or else they’ll come here for a lunch. It's not full of excitement and I’m not a shopper so I don't go out to town for regular shopping I don’t enjoy that. So I suppose it’s quite simple, a simple day-to-day existence. But I also haven’t mentioned I’ve got four brothers and a sisters, so I’m lucky they’re supportive. They don't live near here but I do see them every few months and I speak to them regularly. So their very supportive and helpful which is good. I think that’s about it really.

**CL- Ok. What is your experience of anxiety?**

Clare**-** Well if, if I'm going to go somewhere at a certain time I’m always ready ages before her and, and recently I have found myself I'll be sitting here half an hour beforehand just twiddling my thumbs. And it's just not being able to, not being able to settle to anything and I know my daughters have said to me occasionally I’ve been round at one of their houses and I find myself stood in the middle of the room just hovering. And they’ll say’ mother what's the matter’ and it just feels like I don’t know what to do with myself. So I’m just loitering. Whereas before I’d be quite happy just sitting. If I’ve been there to eat I find it difficult to relax afterwards. I need to get up and come away again. It's like I'm on a mission. I think that’s, well the girls will definitely agree haha!

**CL- So how does anxiety affect you?**

Clare**-** Well it makes my tremor more pronounced for a start. And I just feel, tense. I can feel my shoulders tensing. I feel tense all through. I found, and I found my thought processes go go, go out the window and I’ve always had quite a sharp brain, well at least I thought I had quite a sharp brain. But if I'm anxious or if I’m put under pressure logic goes out the window.

**CL- Can you tell me a little more what you mean about that? What you mean by your thought processes go out the window?**

Clare**-** Yes I have always thought I was quite a clear thinker but if I’m pushed, or if I’m stressed now, I just, I forget basic ordinary things. And I just can't, I just can’t think logically I need to wait until I settle. And go back to a problem. Actually what I didn’t say to you I did volunteer, after I retired I volunteered at the hospice. Do you want a top up of tea?

**CL- Oh no I’m alright thank you.**

Clare**-** I I did about two and a half years volunteering at the Somerset Hospice because I felt, y’know I’ve got all this time and I need something to do. So I was on their front desk the reception. And I did a morning, morning every other week. And, and it was really good I enjoyed it but I was, I had started to find that even silly things like if I had two or three people waiting at the desk. And then, and it wasn't a terribly busy place but obviously it is a lovely emotional place, a very caring environment. There’s a little shop there which I was in charge of and I found if I had a couple of people waiting and another wanting to buy Christmas cards or whatever it was, I would just get in a flap. I’d get my knickers in a twist. Whereas I knew there was no reason to, like if the phone went as I was talking to somebody, whereas before it never bothered me. And I just… that's an example of how my thought processes just didn't work properly. And I just panic and just not deal with it properly. I mean I didn’t make any mistakes or anything but I just knew that I wasn't, I wasn’t coming across as professionally as I should have done. I actually stopped doing the job because, in fact my mother who also lived up the road, she was she was diagnosed with leukaemia last Christmas and during the summer she was becoming more and more frail. And I knew that my connection with the hospice was a little bit too close. It was a bit too close to home so a month or so before she died, and we knew it was imminent, I stopped I had to say to the hospice you know I really don't think I’m managing very well it's a little bit too close to home. And I'm sure she realised because she’d seen me in a dither haha. So and they were fine about it and they were quite happy. But I stopped, I think it's probably about mid-May. So I haven’t replaced that so that’s an example of my lack of, lack of logic.

**CL- Can you describe for me how anxiety makes you feel?**

Clare**-** Well I get sort of, sweaty. And I feel, I feel I go starey. A bit like a rabbit in the headlights. Sometimes. And I just feel that I’m there but I’m not part of what's going on. I feel like I’m an outsider looking in, an observer of a situation rather than being part of it…..

**CL- Ok thank you. How do you react to anxiety?**

Clare**-** Actually, you-you-you, I probably get more anxious haha! I try to slow my breathing down and to relax. I have done mindfulness course as well.

**CL- How did you find that?**

Clare**-** Well I found it quite good. I found out about that through the online thing, the research, where I found you. It was good but I don't think I applied myself, I don’t think I committed myself as much as I should have done. So it's still an ongoing. I got I’ve got the CD and it’s, in fact since Christmas I've been doing it a little bit more as I think I think it's something you need to put more time into. And I think it I think it does help just to concentrate on your breathing and settling down and to be able to get your thoughts together. So, so that's something that I'm ho-hoping to continue with a bit more. It was a Greek girl and she was lovely. It was during this last summer I think it was for 10 weeks we skyped, a group of us skyped every, every Monday night. And we had an hour session skyping each time and that's when we started doing the CD as well. But obviously the 10 week course was just a trial to get us into it. And I’ve still got the booklet and the CDs and think it's up to us now to keep going. I’ve been a bit slack but I haven’t dismissed it.

**CL- Is there anything else that you find particularly helpful for reacting to anxiety?**

Clare**-** I think getting out in the fresh air and playing tennis or going for a walk. I think doing something physically. And I've never been one for the sat inside all day. Although it doesn't sound like it as I have been sat inside quite a lot recently ha! But no I do like the fresh air and doing things and keeping-keeping busy and I do feel better when I’ve done something and achieved something in my day instead of just mooching around.

**CL I really appreciate the time you’ve taken for this interview. Is there anything else you think would be helpful for me to know?**

Clare**-** I think we’ve covered all the main things. Yeah my antidepressants have been upped. Two years ago, I wasn't sure whether, or my doctor and I had a discussion whether early I needed to up the dose of the codopamine, cobenyldopa. But he suggested that my anxiety was more anxiety, not so much the Parkinson's it was, so he suggested that we just up the level of antidepressants which was logical. And I think he was right.

**CL- You mentioned earlier in the interview that about four years prior to your diagnosis you became quite anxious, and on reflection you perhaps attributed that to Parkinson’s. Can you explain a bit about that and why you thought that?**

Clare**-** I think it’s through talking to other people, and with the consultant and the doctor. And what I've read online and what I’ve tried to find out about Parkinson's and it just seems to be common, a common occurrence in in Parkinson's sufferers. And it's just odd that I was never prone to depression or anxiety all my life and it just seems weird that it’s just the last few years that it’s come upon me. I mean, I could well be wrong but it does seem that an awful lot of people with Parkinson's are on antidepressants as well for similar reasons. And we’ve got a little, there’s an unofficial group in <place>. Just a couple of people I know who’ve got Parkinson's so we meet. We meet every now and then and talking to them, and it does seem quite, quite a familiar problem. Just insecurity. I'm not, not major full on depression but just general worrying.

**CL- What’s your experience of having a group of people to talk too?**

Clare**-** Well in fact I don’t really like it haha. I’m fine one-to-one and I'd much rather talk to people individually. Um, I’m not very happy within the group, I mean it's not a huge group there’s usually only five or six of us but it does get a bit monotonous. And that they are all older than me and it’s a bit like being at a pensioners lunch haha! Which sounds very negative and horrible, but there’s a lot of repetition and people are very forgetful which is again then something else that is probably typical. I’m a bit impatient of things like that so I can’t say that I enjoy, and I go along because I feel obliged to, I think we ought to support each other.

**CL- How did you find your experience of the skype group sessions?**

Clare**-** Yes that was ok actually. Yes. Strangely I didn’t mind that maybe because I was here on my own just with my computer. I think some days there were four or five of use, possibly six at the most. But I didn't find that, I didn't find that so stressful which is weird, yes.

**CL- Ok, so the other group, do you find that has an impact on your feelings of anxiety?**

Clare**-** Yes. It will definitely get my tremor going and I just want to get up an out. I must admit that I can sit for an hour or so and then I’m ready to, and I often find an excuse you know ‘I’ve got to be going now’ and I probably haven’t which is a bit, a bit rotten. But I find my tolerane, well I’ve never been overly tolerant anyway I suppose ha, but my tolerance levels have gone down. So I’m less able to to deal with stressful situations.

**CL- And why do you think your tolerance has gone down?**

Clare**-** I don't know really, and I find it with my grandchildren as well. My oldest daughter has got three boys and they’re quite boisterous. I mean they’re adorable but they are full on. She’s got four-year-old twins and a seven-year-old so you can imagine they’re a little bit hyper to say the least haha. And I can only deal with them for short periods of time. If they’re on the case if they’re wild, I just have to get up and go which is, I wouldn't have done that before.

**CL- And what is the reason you have to get up and go?**

Clare**-** I think I’m frightened I’ll say something I regret haha! And I don’t want to get crosswith them because they're not really naughty but they are very lively and boisterous.

**CL- Is that boisterousness of others, does that cause you to feel anxious or is it something else?**

Clare**-** No I think it’s the boisterousness, yes. And I think I feel because I was a teacher I ought to be able to control it. Because when I was teaching I could control it in the classroom, well generally. Because it was my job and I feel a little bit like I have lost that capacity. And maybe perhaps, thinking about it, perhaps I am getting cross with myself that I can't deal with it as well as I used to.

**CL- So does that make you feel more anxious or is it more you’re getting frustrated with the situation?**

Clare**-** I think I was getting frustrated with the situation, yes. I think. It’s difficult to explain. Cos I don't want to miss out on time with the grandchildren but I don’t want them to experience me being bad tempered either. I don’t want to be a crotchety old granny. Have you got children?

**CL- No. I like children but I don’t have of my own.**

Clare**-** Yes well I can understand that hahaha! I like them but it’s lovely being a granny, being a granny is good, you get the best bits.

**CL- Do you have any other questions for me?**

Clare**-** I don’t think I do really.

**CL- Ok. Would you like a summary of the findings?**

Clare**-** I could do, yes, that might be quite interesting.

**CL- Would you prefer them via e-mail or via post?**

Clare**-** Oh, actually by post would be quite nice as I haven’t got a printer.

**CL- Thank you. I have all of the information I need. I will now stop the recording. Thank you.** <recording stops>
