# Supplementary material for: What is the lived experience of anxiety for people with Parkinson’s? A phenomenological study
Source: PLoS One. 2021 Apr 8;16(4):e0249390. doi: 10.1371/journal.pone.0249390 (PMC8031398; doi:10.1371/journal.pone.0249390)
Supplement: S5 File — (DOCX) [file pone.0249390.s005.docx]

**CL- My name is Chris Lovegrove and I'm doing this research Plymouth University. I would like to ask you some questions about your background, your condition, some experiences you have had and about you. You do not take part if you do not want to. I hope to use this information to develop more anxiety interventions specific to people with Parkinson's. The interview should take about one hour but it may be a little shorter or longer. Please feel free to ask for any breaks that you might need during the interview. Are you happy to continue?**

Daniel- Absolutely.

**CL- Let me begin by asking some questions about where you live, what you get up, to your family. Are you still happy to proceed?**

Daniel- Yep.

**CL- Lovely, so can you tell me a little bit about yourself.**

Daniel- I recently retired. Ran my own business, in Sidmouth, which was a family business, four generations old. Parkinson's basically, made me make the decision to sell the business, which was recently only a few weeks ago to be honest. And to retire because I felt if I could remove as much stress from my life as possible it would help. Um quite a hard decision to make because a lot of the people, ‘twas about 70, had been working with me a lot of my working life and my fathers working life. Fortunately we found a local buyer, um to take the business hopefully to bigger things. So I recently retired and I still have quite a lot to do with a property portfolio that I own, own with my business partner. And so retirement isn’t sit down on bottom doing nothing it’s actually doing quite a lot of work. Um try to keep myself active try to do um, and hours worth of exercise per day except at weekends and that includes yoga, specific Parkinson’s exercise classes that we’ve put together ourselves amongst a load of Parkinson’s sufferers and swimming. Day to day is basically enjoying retirement. Doing plenty of cooking which is not good for the waist. Particularly enjoy bread making. Um and trying to get the best out of life as I can and I’m in the fortunate circumstances that I have sufficient funds to enable me to do that… **CL-OK. How old are you <name> if you don’t mind me asking.**

Daniel- 59.

**CL-And how old were you when you were diagnosed?**

Daniel- Two years ago.

**CL- And could you please tell me a little bit about your diagnosis with Parkinson's?**

Daniel- OK <clears throat>. I was experiencing a-a sore shoulder and I couldn't get rid of it with physio I used to go to the gym quite regularly, and couldn’t do bench presses or anything like that. My right hand shoulder was very sore. Physios couldn’t do anything. The doctor was at a loss so he said right let's try a neurologist. So I got in to her see a consultant in Exeter called Dr <name> and within about two minutes of arriving in his office with my partner, um he said you’ve got Parkinson's. That was following a few exercises and walking up and down corridors and studying my gait. Um and that was it I had Parkinson’s. Um I thought Parkinson’s was a TV chat program , but uh haha **<interviewer shares laughter>.** In fact knew nothing about it but soon learnt quite a lot about it. And yeah, I was pretty upset but I’m the sort of type of guy that just get’s on with things so. So just get on and make the best of life you can. Soon met lots of other people with Parkinson's. Sidmouth, being a sort of elderly retirement area, there’s a sort of hotspot of Parkinson's in this area. Er but most interestingly my ex-sister-in-law opened up a "Young persons with Parkinson's” exercise group for younger Parkinson’s suffers throughout the south west.

**CL- Ah right.**

Daniel- Erm which I joined that in Honiton. Which is still going strong and then I became aware of the local group then I’m heavily involved in that..

**CL- Ok. You mentioned about when you were diagnosed and it took a couple of minutes once you met the consultant. Can you remember any more about the diagnosis process, how it was delivered, how it made you feel, was there anything of the particular note that you remember from that?**

Daniel- Well Dr <name> is a very down to earth guy from what I understand. With a good sense of humour so, um, you take it on the chin really. Yeah it was it was worrying. I suspect it's more worrying for the carers. Especially as Parkinson’s gets worse, you know it’s a damn sight harder for the carer than the sufferer half the time. But yeah it was it was delivered in a matter-of-fact way which is just the way I personally would’ve wanted it.

**CL- Right. You mentioned that you learnt a lot about it. I was wondering if you could tell me what the reason for that was**.

Daniel- What learning about it?

**CL- Yeah.**

Daniel- Well I deliberately didn't go on the Internet search too much on. I basically read a few articles, and there’s more articles in the daily press now that you see. But also there’s a lot good articles that come out of Parkinson’s UK. I felt the Internet trawl would just be depressing, cos it's not very good at delivering news on care or illnesses I don't think so I avoided that, and really went word of mouth and talked to people. I went to variety of lectures in Bath which, about deep brain stimulation like that Just really tried to inform of myself the best I can…

**CL- Ok. Could you please tell me a little bit about your typical day.**

Daniel- My typical day right I get up about quart to half past seven. If it’s a swimming morning on Tuesdays or Fridays I go to the pool which has only about 300 yards away. By about 7:30 exercise for an hour and then come back here. I will then I will do some housework allotted to buy my partner. Or I will go and do some work, on my other businesses and then basically I will try and get half an hours kip at lunchtime. So I find half an hours break after lunch helps. And then do something, if I’ve got nothing to do I’ll go for walk. Sidmouth’s a very social place, I know lot’s of people. So generally poodle about.

**CL- You mentioned about sleep in the context of you have a kip sort of around mid-dayish. Could you explain a bit more, how sleep helps you.**

Daniel- Well I think it just refreshes me. Interestingly enough when I was working I was probably having about an hours sleep a day. I owned the business I could do it. So I’d come home have lunch, fall asleep and wake up about 3 o'clock. Interesting enough since I’ve not been working and the stress, a lot of stress has disappeared, um, I don’t seem to need as much sleep so half an hours sleep just rejuvenates me. Er and allows me to get on with the rest of my day.

**CL- So that's quite normal thing for you to do before you were diagnosed?**

Daniel- No, no-no none I was, before I was diagnosed I knew there was something wrong. Something just didn’t feel right and I was just flat out working. Then I was diagnosed with Parkinson’s so I started to wind my hours down. But I found running a family business is quite, stressful sometimes. Especially it was a contracting business dealing with the public and contractors. So you had stressful days you had non-stressful days. A sleep would just help rejuvenate you.

**CL- Ok. Do you find that sleep particularly helps the symptoms you experience? Do you notice things are different if you don't say, have a sleep at midday for example?**

Daniel- Erm, no I don’t think I do actually to be honest, just feel rested. The Parkinson’s doesn’t appear to change in anyway.

**CL- Ok. So what is your experience of anxiety?**

Daniel- Erm, I get anxious, take for instance I went to watch Exeter v Saracens at the weekend at the Exeter rugby. I got anxious about finding the seat. It was really strange statement I’ve not really had that before. It was a strange stadium I didn't know where I was going. I was anxious of the loss, I was by myself and I was anxious about where my seat was. As it was I was very lucky it was probably the best seat in the house right next to the head coach of Exeter! But that’s another story. Queuing for drinks at the bar when busy, when there’s lots of people around I get anxious. At the airport going abroad, anxious about being challenged about what’s in your suitcase, you just worry about things more than you did before.

**CL- And is this something that you wouldn’t have worried about necessarily in the past or you’re worrying more so now that you’re diagnosed?**

Daniel- We all go through these dreaded research things at airports, it’s just it’s just more anxious with Parkinson’s. Your thought processes isn’t as quick and you get asked questions stall.

**CL- That’s interesting you mentioned because you knew you don't answer as quick and you stall and things like that, do you think you worry more and are more anxious because of things like that? Or do you feel anxiety perhaps it is a symptom of the condition itself? What would your thoughts on that be?**

Daniel- Erm my limited Parkinson's experience being only two years down the line, my anxiety has increased without a doubt. Stupid things, like if I think in the middle of night, y’know, I ‘ve got to do something and get you get anxious about remembering to do something. I’m waking up at four in the morning thinking I mustn’t forget that, I mustn’t forget that…

**CL- So how does anxiety affect you?**

Daniel- I think it makes me slower, speech wise it-it appears to change my gait when I tired and anxious. My gait changes, my walking pattern changes. To small steps. That's probably it if I get anxious, if I get tired that happens as well. So, so they seem to be linked somehow….

**CL- Are there any other ways it affects you that you particularly notice?**

Daniel- Erm. Not really it’s slowness of slowness of movement and slowness of speech and it seems to slow down. The more anxious you get the slower it gets. **CL- Is there anything you find helps when you’re feeling anxious or helps you to overcome that?**

Daniel- I think I'm very lucky with my partner <name>. Whose grandad died, or had Parkinson’s when he died. Erm so she’s experienced it and will jokingly tell me don’t be so stupid and buck up you know. She kicks me up the bum know and then if I'm feeling anxious or worried about something I shouldn’t be worried about.

**CL- Do you find that’s quite an important thing having somebody else give you that pick up?**

Daniel- Absolutely, absolutely. It gives you the pick up and gives you the confidence to do things…

**CL- Ok. You mentioned when we were talking in the kitchen before the interview about the exercise classes you’re involved with. One things you mentioned that was a group of other people sharing in having Parkinson's and I was wondering if you can't explain a little bit about that? That sharing an experience with the people and if that’s helpful for you.**

Daniel- I mean yeah very much. Parkinson's is a big neurological disease. No one knows how it’s going to pan out and there’s a certain amount of nervousness about it. But if you’re with other people who are similarly affected you can discuss, talk to themselves, their carers, their wives, their husbands. There’s a few people younger than me not many. And we share experience. To be fair you become very friendly with them. Quite often people will come here for dinner and we will go to other Parks sufferers for dinner. So it’s almost like a separate community really. Erm. And it’s very strong and it’s growing We just had some people moved down here to the area from up North somewhere and they come along to the exercise class and met a lot of like-minded people with Parkinson's. It’s gotta help.

**CL- do you find that that shared experience and sharing with others, does that help your anxiety at all or does that anyway make it worse?**

Daniel- I mean sometimes summin, I went to, as treasurer I went down to the other exercise class in Exmouth that’s run by the same group. There was a lady whose whose husband’s bed ridden with Parkinson’s. You do take a reality check when you hear that. That that made me quite anxious that conversation. I could hear myself thinking, bloody hell. But then you’ve just got to get on with it and there’s always someone worse off. The great thing about these groups is a lot of them have had Parkinson’s for quite a lot longer. There’s a few people in their 80’s. Some have had it for 10 or 12 years and some have had it for 50. But they’re still able to go out, to quiz nights and erm. If you’ve ever seen a load of Parkinson’s people ever play skittles that is really quite funny.

But everyone laughs with each other which is a really great thing.

**CL- Ok, thank you. Can you describe for me how anxiety makes you feel? So, when you're feeling anxious and you’re having one of these episodes.**

Daniel- A sort of compression really I think. You feel everyone is watching you, you’re looking around. Lack of confidence I think, perhaps comes in with anxiety as well…

**CL- Are there any other feelings you ascribe to it, perhaps if it is affecting your gait or anything like that? Are there any other ways it makes you particularly feel?**

Daniel- It’s gait and just slowness for me. I feel the pressure’s coming in on me. My gait changes and my thought process slows…

**CL- And how does that feel for you when your thought process slows?**

Daniel- Like if you’re in a supermarket and someone tells you where something is, you think to yourself right I’ve really got to remember that. You go up the aisle and you try and find it. I was in B&Q last night and the guy, it’s the third aisle up on the right. So you think right, it’s the third aisle… then you ten steps and you think was it the second or third aisle? Anxiousness takes away your ability to think straight. Or it does for me anyway.

**CL- Thank you. When you have that experience of your losing your ability to think straight is there anything you do you find helps?**

Daniel- Not-not specifically. Get the task you’re supposed to be doing as quickly as possible. I think… probably going back to the rugby I didn’t know the ground I didn’t know the park. It’s like coming home you know where everything is you go into someone else’s home and you’ve got to find things. It is more difficult. Recognising and being in the same place things position is helpful.

**CL- So how do you react to anxiety?**

Daniel- I can I get tired and therefore, I try to have a rest or sleep and try to put my feet up for an hour if I get particularly anxious about something. Or sit and read a book, or read the paper, sit and do something peaceful. That seems to sort it out…

**CL- Your experience of your exercise you’re a part of, how do you feel that has affected your anxiety if at all?**

Daniel- Erm, it’s probably improved, I’m probably less anxious because I know there’s like-minded people out there I can share the experience with.

**CL- Ok…**

Daniel- I remember first being introduced to these people, both my partner and I were pretty anxious as we didn’t know what to expect. Familiarisation, so if somethings familiar you know the pattern and what’s going to happen, so you you don't become anxious. If it’s something unfamiliar then anxiety strikes.

**CL- When you say anxiety strikes, is it a slow creeping feeling or is it sudden, is it quite fast?**

Daniel- I think it depends on the circumstances, if you are walking through an airport checkout you become anxious. If the guy says right I wanna search your bags. Then, merrr, my Christ. So so I think anxiety for me can be a result of depending on the circumstances how the anxiety hits you.

**CL- So the speed of how it strikes is dependent on the circumstance.**

Daniel- I would say so.

**CL- Ok. That's really helpful thank you. I really appreciate the time you have taken for this interview. Is there anything else you think would be helpful for me to know?**

Daniel- Erm. I don’t think so. Shame we didn’t discuss that question at the beginning I could’ve given it some thought. Not right off but I might come up with something in a minute.

**CL- That’s ok, I’ve got a another question that will give you a bit of time to think. You mentioned at the beginning, when you were learning a lot about it you avoided particular Internet resources and you found things like Parkinson's UK, you used their resources. Did you find that learning more about your condition, did that help your anxiety or did that make things worse?**

Daniel- I think I think to start with it’s bound to make things worse because you realise you’re ill and it’s an incurable disease at the present. So you you you you you’re facing an uncertain future but again coming back to these groups, if you’re with a load of like-minded people you’re equal to them so it's a great leveler really.

**CL- From how you described accessing different resources it sounds like your quite computer and tech savvy. Do you use many Internet resources? You talked about group experiences and sharing with others, do you use many of the other resources that are out there, for example the Parkinson's UK forums or anything like that? Do you make use of those?**

Daniel- Erm I don't to be honest I prefer with something like Parkinson’s to talk to people face to face. Erm it’s like Facebook I don’t do Facebook, Instagram and Snapchat and all those, although they are very valuable. I use email a lot. What can’t you do on the Internet nowadays. It makes life a lot easier buying things, banking things like that.

**CL- That's really helpful. I think that’s all of my questions have you noticed that you wanted to ask?**

Daniel- So how are you just out of interest, you’re going to get a lot of people completing these things what are you hoping to achieve?

**CL- So my aim is, as occupational therapists I have worked with lots of people with Parkinson's who experience anxiety as a crippling symptom of their condition. What I found in my practice is we have lots of specific treatments for all manner of different things such as freezing for example, all manner of different symptoms. There is not really anything out there to supporting people with Parkinson who experience anxiety. So the quite often doctors will try pharmaceutical treatments but actually in Parkinson's the evidence for them isn’t hat good. Some of the work is happening at the moment for more directors so-called behavioural interventions are taking pre-existing interventions for people who don't have Parkinson's. I described it as make a square peg fit a round hole, they’re trying to make it fit the condition. My purpose for doing these interviews is in the future, a few years down the line, is to start developing a Parkinson's specific anxiety intervention but one built from the ground up of people with Parkinson's experiences. So that where this work is leading to. That's I'm asking people about their experiences, what it means to them really because this work hasn’t been done. So that’s where we are going with it.**

Daniel- Yeah I mean there’s a lot of work happening with Parkinson’s it just needs some more money really. It’s the poor relation to cancer as far as fundraising appears.

**CL- It's interesting you say that because that's been mentioned by other people. Is that a, is that a feeling that you have generated yourself or is that something you've noticed looking online or…**

Daniel- Well generated myself really. If you look around charities and the money that’s applied to cancers by its very nature it’s huge amounts of money compared. Parkinson's is a relatively small but you know, again that’s part of this group looking to fundraise, giving grants to local authorities. We’ve just got a grant from active villages Devon, which is to keep all the villages active so we applied for it, got it, and what we’ll use that for is to reduce cost of the exercise classes.

**CL- You’ve talked a lot about being involved in exercise and social groups, and there almost like another step forward in being engaged in social activism and community engagement. Do you find that’s something that helps with your anx-**

Daniel- Absolutely yeah. What we've got in the group unfortunately are the well-off Parkinson’s sufferers who can travel, who can afford to do these classes. My feeling there’s a whole host of Parkinson’s sufferers out there that don’t even come outside the door have the time, they can’t afford to go to these classes. That’s one of the reason’s we are trying to up the ante on the fundraising to see if we can if we can make them virtually free. The more we can keep people exercising a physically fit the less they will be in hospital which costs the NHS money.

**CL- Thank you. Have you got any questions for me?**

Daniel- I don’t think so Chris.

**CL- Was there anything else that you would like to add at all?**

Daniel- Nope, not at this moment in time.

**CL- Would you like a summary of the findings?**

Daniel- Absolutely.

**CL- Thank you I have got all of the information I need. I will now stop the recording.** <recording stops>
