# Supplementary material for: What is the lived experience of anxiety for people with Parkinson’s? A phenomenological study
Source: PLoS One. 2021 Apr 8;16(4):e0249390. doi: 10.1371/journal.pone.0249390 (PMC8031398; doi:10.1371/journal.pone.0249390)
Supplement: S6 File — (DOCX) [file pone.0249390.s006.docx]

**CL- Okay my name is Chris Lovegrove and I'm doing this research of Plymouth University. I would like to ask you some questions about your background, condition some experiences you have had, and about you. You do not have take part if you do not want to. I hope to use this information to develop more anxiety interventions specific to people with Parkinson's. The interview should take about one hour but it may be a little short for longer. Please feel free to ask that any breaks that you might need during the interview. Are you happy to continue?**

Edward- Yes.

**CL- Let me begin by asking you some questions about you, where you live and your family. Can you tell me a little bit about yourself?**

Edward- Um right my name is <name>, I’m retired from the IT industry, project manager, salesman over 30 odd years. My wife and I retired to Wadebridge about five years ago. My wife was also in the IT industry. Uh we have two adult children. A daughter living in Reading and a son living in Sydney. Uh I think that’ll do for an introductory paragraph.

**CL- Could you tell me how old are you at the moment?**

Edward- Uh was I’m 68.

**CL- And how old were you when you were diagnosed with Parkinson's?**

Edward- 62ish.

**CL- Ok.**

Edward- I’m deliberately vague as there was a prior diagnosis of essential tremor. Which I was assured at the time was not Parkinson’s but in the benefit of hindsight clearly in was a step along the way. So if you take that into account then I go back to about, about the age of 58. To the earliest symptoms. So 10 years ago.

**CL- Could you please tell me a little bit about your about your diagnosis of Parkinson's, and what I mean by that is the process and how it happened for you.**

Edward- Um… how to summarise it. I didn't find the process particularly earth shattering which I guess many people do, because I guess all the prior diagnosis of essential tremor prepared me for physical aspects, some of the physical aspects. And from a, from a mental point of view I suppose I’d been relatively stable and sensible since the get go. So when I came to be diagnosed, and this was after we moved to Wadebridge, I suppose it didn’t come as a huge shock at the time. Um the consultant handled the process reasonably well. I’m certainly not the victim of the horror stories that one, one hears and the people I now meet quite regularly. In a social context. So I suppose for me the diagnosis was a natural significant step in a process. And um, as I’ve discovered the truth that your journey with Parkinson’s is a very individual one. Until relatively recently, until the last few, I've been free of any significant symptoms and it’s only in the last year or so that it’s begun to get in the was of normal life. Um so, not earthshattering, are not, it hasn't caused me significant depression, indeed I’ve no-not become clinically depressed at all. I’ve become a bit grim at times as my wife will tell you. But normally perhaps because I’ve found things to do, I’m diverted by things I do. I really don't think about it a great deal but having said that as you’re well aware from working with people with PD one is always aware of it. When you wake up or when you’re going to sleep, whatever you do, there’s a cue which reminds you ,you , you have Parkinson’s…

**CL- And what sorts of cues are those?**

Edward- Pain, discomfort, in my case freezing of the gair. Tremor when in an off period. possibilities These are the physical cues which, which, again are common to the condition…

**CL- Okay thank you can you please tell me a bit about your typical day?**

Edward- What in the context of PD symptoms or?

**CL- Just what you do in general really.**

Edward- Well all most of what I do to to keep me useful is desk based. Since I’m a refugee from the IT industry I’m fairly competent with computers and with the applications. So I do a lot of work for myself and for others with the computer. I also had, I also struggle with the negative effects of a bad hip revision about three years ago which gives me quite a lot of pain when walking or standing for any period so I'm kind of directed towards sedentary occupations. So the normal day will be waking for the first dose about seven or usually later because I stay asleep a bit too long. We have very leisurely get up and go process in the morning, so breakfast is generally not until ten, ten thirty. And then my wife will go off and do the shopping and I’ll be left doing whatever I’m doing. I recently joined the gym which is 200 yards down the road. So I go there two or three times a week for at least upper body exercise. So I’ll typically be out for that for an hour. If we do if we socialise we, we prefer now to socialise during the day lunchtime rather than dinner, particularly if I have red meat that soaks up the levodopa. So diet and timing of meals is something we need to be careful about. So we we will socialise in the afternoon. Because we have a late start to the day lunch is always late. Um if we have meetings we may have a social meeting or we may have a Parkinson's club meeting of some description. My wife and I belong to Parkinson’s UK, we belong to a couple Parkinson's support groups locally. We’ve made several friendships through the medium of these groups. So I’ll either go and support one of the groups and give a talk or something like that. Or we will just go and meet them socially which is something we have planned for tomorrow in fact. The evening cycle. Well my wife is learning to play the accordion soI’m supposed to be the timpani accompaniment haha! So she’ll typically practice after lunch for an hour or two. We agree she shuts the door and I go to the other end of the house haha. I mean that’s not a comment on her playing it’s just that there’s no need for me to sit in the room. So that brings us to the early evening, then watching the telly. Almost always was recorded rather than live. Then, to bed. Well, now we now go to bed separately as we’ve found, I just have to get up and I’m restless. So I will now generally do puzzles or whatever until about midnight and that will generally see me off. By the time I get to bed then at midnight I’m off.

**CL- Do you find that when you go to bed that you can sleep right the way through?**

Edward- Um no I don't sleep right the way through either the pain in the thigh or the bladder will get me up. Typically once in the night. So I will typically wake somewhere between two and four. Um if I need to get up I get up. Um I go back to sleep almost straight away. So the duration of the sleep is shorter than it was but I have no problem going to sleep should I need too. Dreams, yes, almost always of work. Which is a bit disappointing. Sometimes, sometimes unpleasant but not nightmarish. Only very rarely is it nightmare proportions. I don’t thrash around so we still sleep in the same room. Quality of sleep, well I say I go to sleep quickly but I’d say the quality of sleep is pretty poor. By my, from my previous history as it were. I used to sleep pretty well. Now I toss and turn a bit and I snore, or so I’m told. So and that brings us to the morning where I suppose pain and stiffness is the waking cause. And most recently a lot of pain, well and a lot of stiffness. Once I get the limbs moving I’m ok.

**CL- You've mentioned about your, your Parkinson's symptoms and have touched on those with timings of meals for example. Are your Parkinson’s symptoms each day generally stable or can they fluctuate?**

Edward- They can fluctuate and while I would say for the large part when I'm moving when I’m mobile it’s predictable. So at the moment the drug cycle is seven, 12 PM, five and 10. I can predict we can be reasonably clear I can be reasonably clear in that given time when I will be moving freely and when I’ll start to have difficulties. Given I’m 45 minutes off the next dose I'm beginning to beginning to find it difficult to move around. The hand expressions are beginning to falter. Having said that it can fluctuate not violently in the sense of unpleasant but normally I find it very difficult to move towards the ten o clock regime but somedays I can be moving entirely normally as if nothing ever happened. Just occasionally a lightswitch comes on and poof, we’re off and moving. As you can see I measure my symptoms almost entirely by physical issues. I find no particular metal cycle. I don’t find and attention deficit. So so predictable mostly but when it's when it goes unpredictable it’s ridiculous. It goes completely the other extreme.

**CL- Do you find when it goes to the other extreme does it take you some time to get back on track so to speak?**

Edward- No. No I mean it could be, if I’m unpredictably up and going it can be for half and hour or so. Then it has gone. Similarly if, sometimes this morning it took 10 or 15 minutes longer to get going. But only 10 or 15 minutes. So I’m not, if the cycle if the pattern goes off it’s for a short period. It’s not for a whole day I’m not, I’m not I’m not frozen for a whole day. Or moving for a whole day.

**CL- When you have those patterns how does that make you feel?**

Edward- My thoughts are of frustration. Um. And an intellectual curiosity as to why, as to why should why it should be like that. I mean I have some understanding of the clinical causes as to why it should be like that. Uh the clinical situation but that, that of itself can not explain why suddenly the lights come on and you’re moving for half an hour. Whereas typically you don’t. Um thinking to the theme of your study, does it of itself make me more anxious than I was. No. The event the event, the events don’t make me anxious they make me angry. Angry with a small a. There’s no point me getting angry with a big a because nothing’s gonna change.

**CL- So** what is your experience of anxiety?

Edward- I would say of two levels. Anxiety about, well.. I suppose the simplest way to explain it is to go back to my career which was most of which has been spent in sales. All of which has been spent on frontline of IT. Working for suppliers on contracts and project managers, sales campaigns or whatever so that's always brought me into, that’s every day in every way brought me into contact with with other people with new situations, with trouble. Um and nowadays I’m less able to cope with that. Much less able to cope with that. Much less able than I was. It makes me, it makes the whole process, the whole process makes me nervous. And I suppose to paint the picture I for the last five years or so had been treasurer of a local vegetable growing enterprise. A community interest company. A it became apparent to me a year ago as treasurer counting the numbers, that this enterprise was in serious danger of going down the tubes. And because of the circumstances surrounding its inception and it’s current performance I could see that the exit, well a, the exit strategy had to be determined. And b it had to be performed. And I decided quite, as pretty much as soon as I recognised, well as soon as I predicted what was going to happen. That I was not going to be able to handle it. Whereas 10 years ago it would have been <ineligible> to find a new site, to negotiate new sponsors. All that stuff. I thought no, no I will not I will not be able to contribute. It'll make me nervous, anxious, I’ll find it will be difficult for me to enunciate my thoughts. The logic will be there but ability to deliver it will be constrained, um if not ineffective. So I decided to take myself out of that situation. So so what are, that's what anxiety means to me at one level. So I now make sure, the way the way I engage with the outside world is predominantly in social context now. If I give a talk to my probus group or my the PD group, it’s low profile it’s not a big issue. It’s something I’ve done before, it’s something I can rehearse. It’s something I can be very comfortable about before I go to the situation. So I guess I I get anxious about a situation which, which contains risk of risks. Of unknowns. I say something about the process by which you set up these interviews, all this is clearly designed to to reduce the anxiety issue, because you get letters about letters you get calls about calls. Then you get a call, then you get another call. So clearly this is all designed to damp down, well if it isn't then that’s what it’s doing!

**CL- Absolutely, absolutely. I ran a consultation with people with Parkinson's who also experienced anxiety to see how they would want a study of this type to be conducted. So this has come directly from people with Parkinson's.**

Edward- So that's that’s one level level the other I suppose will be the much more intellectual level of anxiety about the condition and where it might take me. And those who, well my wife is increasingly in role of carer with a capital c rather than a small c. And that’s an anxiety which is, it is always there but I would say up to now it’s background. Background noise. I can see depending on the way the condition goes I could be more and more anxious about me and those around me. But I hope to hang on to the fact that it’s the way it is. Until, until we get past the condition with medications which no more than alleviate, we are where we are. I try and assuage that anxiety by getting involved in programs like this. Uh and pretty much anything that Parkinson’s UK can throw at me. <name> can do this, <name> can you do that. Um small grant application reviews. Stuff like that. So I’m anxious about me, maybe I can help, maybe there’s something that I’m motivated about helping others yeah I suppose so. But I’m more hopeful that something that I do in one of these programs will trigger something that brings a solution quicker for me. Even though with what little I know for example about the drug cycle, adoption and its introduction cycle, the chances of anything getting to me in time are fairly small. Repurposing has become a big issue over the last year or so. And maybe something repurposed can at least improve the alleviation and treatment of the symptoms rather than the condition itself. Which will be of some benefit to me. So short-term anxieties to do with meetings and risks, I mitigate that by avoiding it, withdrawing from treasureship ha! Strategic anxiety mitigate by just being involved in things that I can make a contribution too.

**CL- Thank you, so how, how does anxiety affects you? When you feel anxious what are the thoughts and the feelings that you experience?**

Edward- ………. I find that extremely hard to a-answer. I can't I’m, I can’t say I’m conscious of a particular train of thought that happens. There, I’ll be, I guess….. The thing that strikes me most about the a-anxiety is the physical manifestation and I keep coming back to that. That forms a circle because I when I get anxious, the obvious, there’s the obvious tremor. Which is… I suppose in a public situation that that’s a vicious circle. Um so if I start freezing and I’m in a public place, I will freeze completely. If I have a tremor in a meeting situation, that tremor will will not become dyskinesic in this sense <gestures> it can be quite marked. And I’ll need to physically sit on the hand, and think of and do something different think of something different. Do I have any thoughts of about me? I-I really can't I really can't identify any thought trains. The anxieties manifest in a physical way. So far.

**CL- So just that I’m clear I understand from what you’ve just explained. When you have anxiety you particularly notice your physical symptoms become more pronounced. So things like your tremor, your freezing become much more marked?**

Edward- Yes.

**CL- Okay.**

Edward- And clearly there are thoughts, there’s a thought that arise from that situation. Sort of, well, frustration and I think now I’ve finally navigated my way to to to an issue. For example the freezing of gait there are n suggestions where n is a large number of the way in which you can overcome the tremor. One significant way have you seen those before the lasercane?

**CL- Yes you push it down and it projects a line.**

Edward- Right. Absolutely clear how it works and 90% of the time it works as it should. The other 10% of the time it doesn't. And you’re sitting there thinking so how else do I, what other strategies can I adopt to try and get myself moving so, there’s talking to yourself. Counting. Um which is ok in private but I find I find it very hard to speak to myself in public. I even find it hard to speak to myself going down the corridor sometimes. I have a metronome on my mobile but the whole process of getting the mobile out and setting the metronome going, it’s often not appropriate to the situation. And the thought process then gets, in the same way as you I physically stuck I can get mentally stuck. I can only like it it’s not a vicious circle because that indicates motion. The brain stops! And I mean it’s 15 seconds, 30 seconds whatever until you’ve either kicked yourself out of it or my wife will say something or do something. So I guess that that's the that's the mental aspect that is the only mental aspect I can identify of any significance. That I can identify to do with anxiety so we’re saying in the same way the body stops, the brain stops as well. It loses its place. Finding ways to get out of the situation I’m in.

**CL- And when you get that mental stop and that mental freezing, is anything you can do to help alleviate that or do you just have to let it pass?**

Edward- Um you have to let it pass. I mean it passes, it’s not it’s not five minutes it can be five seconds it can be 15 seconds. It’s almost as if the brain has to stop in order to reboot. I don't metally reboot or physically reboot it stops and then it starts again. That's the picture I’ve formed talking to you

I haven’t formed that from before. That's how I would describe the situation.

**CL- That mental stopping, does that have any impact on your anxiety when you experience it?**

Edward- No I mean, I think the sensation would be one of relief. When one is released from from the lock. You get going and, I wouldn’t wish to indicate that I’m particularly nervous about public situations. I say that because I read a lot about people who who who suffer criticism or anger in public situations and therefore react don’t like going out or react badly. I’ve never found that I’ve always found the public to be very considerate. I’ve not encountered anyone pushing me aside or, maybe because I’m standing six-foot tall and I look reasonably well-built maybe. I I I think for a lot of people the grey hair helps. Or what’s left of it. So I don't I’m certainly not afraid of public situations. I don't I don't I I now do things that I never did before. I now strategise about my route through a public place. I’m now looking ahead for the corners and the doors. So I’m looking for situations where I’m at risk for example of freezing in an off period. That's really the only management that I do with public exposure. So I diverted there because thinking about anxiety in public places, my brain stops.

**CL- You’ve already touched on it but I think it’s worth exploring a bit more. How do you react to anxiety? How do you respond to those increases in symptoms, how do you react to that?**

Edward- WelI, aggressively would be is the word that springs to mind immediately so I’ll use it. I have no doubt that psychologists would say positively. But aggressively I I don't like I don't like… I don’t like being like that. I’ve I think in the last three or four years I had one one episode where I broke down and wept for something. I don't remember what triggered it but it was something proably trivial. And I didn’t like the me that did that. And I’ve never done it again. And I hope it stays that way. Because I don't know why why should I think that way because I don't think it's worth it it’s certainly not helpful to the person standing or sitting in front of me because there’s actually nothing they can do about the the situation. Soothing words, pfft, it’s that’s not gonna help. So it doesn't help the person in front of me if there’s someone there, and it doesn't really help me. Some say it clears out the stables it releases feelings, well for me something like that doesn’t. It just makes it worse. So if I get anxious I tend to react aggressively to it. I tend to try, to go and do something else which is what I can do except when I’m in bed and it’s the middle of the night. But as I said I sleep pretty well, I don't lie awake agonising about it. I do things that exhaustive me physically so that when I go to bed I’m ready to sleep. SO all of this is embroidery around the the basic where reacting against it by aggressively and typically by doing something else. Moving away, physically or mentally, thinking of something else. And that's why my to-do list is quite long. And varied. And sometimes the frustration of my wife. Why are you sitting at that bloody computer again or whatever it might be.

**CL- I really appreciate the time you took that interview. Is there anything else you think would be helpful for me to know?**

Edward- Um I’m just thinking of the the people will we, we socialise with. And how their anxieties are manifest. The anxieties it seems to me, maybe I’m a case in point, anxiety is manifest in different behaviours. I’m not going to name and names I just thinking of some of the people I know. I know from talking to them and being with them over a period of some years now that they are anxious from time to time. But how they demonstrate that anxiety is is a behaviour it is not is not sitting there looking anxious. It’s something they do differently. Either things they shouldn’t be doing, or more of, obsessions particularly given the side effects of some of the PD medications. Obsessive behaviours. So how the resolve their anxiety is by obsessively not cleaning the house! Making their life more and more difficult by accumulating more and more stuff. Some it’s a bit too much to drink. So they try to substitute their anxiety with with with doing something different. So I would say look for replacement behaviours. Some of which are appropriate some of which all aren’t. How do people resolve their anxiety. Do you just react by sitting there an doing nothing, or does that make it worse and you end up feeling like you want to kill yourself. After two hours or four hours like this.Or do you go and do something different. Thinking of my own condition and others I know, the anxiety is resolved or not by behaving differently. Differently badly or differently well or just differently different. That’s it.

**CL- Do you have any other questions for me?**

Edward- Yes I’m curious as to whether you see, what do you see that is different in anxiety in PD as compared with those who don't have PD. Because anxiety is a state that everyone experiences. What is it about anxiety that you see to be different?

**CL- That’s a good question. So in Parkinson's we know anxiety is a non-motor symptom a lot of people experience. Some things I’m finding, some people who say I was always an anxious person before having Parkinson’s and then having Parkinson’s it it's become worse. There seems to be a number of people who say I never ever had a problem with anxiety I was always a very confident person until I had Parkinson’s, and anxiety is probably one of the most crippling symptoms of having the condition. They directly relate that back as a a symptom as well as their physical symptoms, it is it’s own stand-alone thing to do with brain chemistry and how that has changed. There’s a number of people who say I experience anxiety, such as yourself, particularly due to when I freeze, it gets worse, in public I get worse. You have that experience anxiety. As opposed to say the population of people who don't have anxiety they don't experience those particular symptoms and they don't necessarily experience those symptoms, like freezing of gait for example. The reason I’m doing this work is that my background is as an occupational therapist, and working with people with Parkinson’s I found that anxiety was a symptom that could be crippling. That was the word people used. But the treatments weren’t very effective. They were either medi-er-pharmaceutical treatments that weren’t very effective. Or they were the behavioural interventions that were starting to be used weren’t overly effective. They come from an evidence base that is being used a population that don't have a neurological condition and they're trying to adapt that for this population people. And the results are kind of mixed. So where this work is coming from, the future aim is to develop an intervention for people with Parkinson’s, specifically who experience anxiety to support that. But building it from the ground up based on their experiences as opposed to trying to, I describe it to people as trying to make a square peg fit a round hole, by trying to take something that works for others that may not necessarily work quite as well for others. So for example some of the things out there have looked at looked at using mindfulness, or yoga or t’ai chi. They have some effectiveness but-**

Edward- They may have been effective with that person if they didn’t have PD.

**CL- Quite, quite. So we’re trying to take a different tact and build really something from the ground up and involving people with Parkinson’s from the very start and developing it from there their experiences.**

Edward-There are lots of neurological conditions unfortunately. There are not many not too many long term neurological conditions I can think of, well diabetes type 1 is one example that springs to mind. Someone with diabetes would be anxious about the condition and what the future has to hold. I’m trying to tease out any the differences between between anxiety about having this long-term condition compared to others. Or is the special case in Parkinson’s about mobility, or is their something in brain chemistry which affects which which does make people anxious.

**CL- So I couldn’t comment for diabetes, looking at some of the other literature that I’ve read around for other neurological conditions. Some of the more shared experiences are around that fear of the future, the not knowing, things like that. Some of the more, what has been touched upon briefly more in Parkinson’s literature, but it's very tenuous,work has touched upon the anxiety umbrella and things around movement and things around not be able to cope in social situations, not being and a burden to others as the condition progresses. Things like that. Those seem to be some of the more specific things a bit not Parkinson’s that I noticed at the moment.**

Edward- Some which of course there is no treatment for. I suppose you can get people to think more positively but that’s about it. Yep, ok, fair do’s.

**CL- Would you like a summary of the findings?**

Edward- Yes I would.

**CL- Would you prefer those via email or post?**

Edward- Email.

**CL- Thank you. I have all the information I need. I will now stop the recording.** <recording stops>
