# Supplementary material for: What is the lived experience of anxiety for people with Parkinson’s? A phenomenological study
Source: PLoS One. 2021 Apr 8;16(4):e0249390. doi: 10.1371/journal.pone.0249390 (PMC8031398; doi:10.1371/journal.pone.0249390)
Supplement: S7 File — (DOCX) [file pone.0249390.s007.docx]

**CL- My name is Chris Lovegrove and I'm doing this research Plymouth University. I would like to ask you some questions about your background, your condition and some experiences you have had, and about you. You do not have to take part if you do not want to. I hope to use this information to help develop more anxiety interventions specific to people with Parkinson's. The interview should take about one hour but it may be a little shorter or longer depending on what you have to share. Please feel free to ask for any breaks you might need during the interview. Are you happy to continue?**

Gillian- M-hmm, yeah.

**CL- Can you tell me a little bit about yourself?**

Gillian- At the moment, I’m just a mess that’s all. That’s all I can say. I used to work, I worked in the doctors surgery for 16-years. I was full of confidence, I didn’t give a dot about anything.

**CL- Right.**

Gillian- And lately I feel like I’ve changed completely I’m a totally different person….

**CL- When you said you you’ve changed, you changed completely to different person. Is that quite a recent change or has that been over a period of time?**

Gillian- Gradually, I’ve lost all my confidence. I have no confidence in anything and that's not me. I mean I’m making his life completely miserable. Yeah I’ve I’ve just changed I I I don't know what what’s brought it all on but it's been a gradual change. And I know all the answers and I know that I should do more. Because the less you do the less you want to do. I've lost confidence to do things you know. I hate it, I hate the way I am. I absolutely hate it.

**CL- How old are you at the moment <name>?**

Gillian- 73

**CL- And how old were you when you are diagnosed with Parkinson's?**

Gillian- Parkinson’s? 70. Three years ago.

**CL- Okay.**

Gillian- But I probably had it a-a lot longer than that.

**CL- Could you tell me a little bit about your diagnosis with Parkinson’s, how you were diagnosed, the process that you went through?**

Gillian- What do you mean to the GP first or the consultant?

**CL-Let’s go back to the beginning to the GP first.**

Gillian- Well I went to the GP because I was once again massively tired. And I know it's not a great thing in the scheme of things but my thumb used to shake. Just you know like that. And I did tell her and she said to me can you control it, and I said yes. I was depressed according to her, I had depression. So she started me on antidepressants which I couldn't take I felt absolutely dreadful on them. After about three or four months we eventually found something that did agree with me but then I wanted to come off them and that was, how on earth coming off them. Eventually I got <partner’s name> to come in with me to see her. Cos I was struggling by then I was struggling a lot. And she referred me to the neurologist and I did see him. And I walked in the door to see him and he just looked at me and said you've got Parkinson’s. And that was <doctor’s name>. That was basically it, he did a few little tests I think did he I can’t remember. I couldn't do this <rapidly turning over hands repeatedly> I can do it now but I couldn’t do it then. Within about five minutes he said to me ‘you've got Parkinson’s’. Here I am. Here I am now, a mess.

**CL- When you were diagnosed, when you had that appointment with the consultant can you remember much about that? How it made you feel what you’re experience of that was?**

Gillian- Well in the way I felt quite relieved that there was something wrong with me that I wasn't actually, going a bit doolally or whatever you know. But then I suppose it all hits home but most people with Parkinson's, <partner> knows a few, and the new consultant I’m seeing now he said most people with Parkinson’s take the medication and their fine and they get on with their life. But I don't seem to feel that I don't seem to be like that. I just feel something is quite right. I don't know whether it's just me or but I don't I think he says the Parkinson’s is under control and I suppose it is. It’s the other things that are making my life so miserable I don’t know. **CL- And if you don't mind me asking when you say other things, what do you mean by that? You don’t have to share that if you don’t want to.**

Gillian- Well the way I feel I don't feel, I don't sleep well which I think is the biggest problem I’ve got. Because you know if you sleep well you function don’t you but I can't function. I just feel… pretty grim all the time…

**CL- So can you tell me a little bit about what your typical day is like?**

Gillian- See where I am now <sat in armchair>. That that's that been about it for the last couple of weeks. I know I shouldn't I know I should exercise I should go out I know I should. And the only time I’ve been out is to go to the doctor, and that's been about it. And I’m ashamed to say it it’s a horrendous way to live and I shouldn't be living like it. **CL- You mentioned that you should be exercised and doing more. What do you think is stopping you from doing that?**

Gillian- Can’t be bothered I suppose. Which is awful to say. Awful. I used to walk miles, miles! I used to walk to work and work all day. I feel unsteady if we do go out I’m always clinging on to <partner>. But I hate being like this it’s not me at all if you knew me before all this, I was a totally different person you know.

**CL- Have you had many falls?**

Gillian- No I only fell once in the kitchen but that was because my blood pressure was so low. When I stood up I just went. Normally I can get into here but I just went but that's the only time I’ve actually fallen. **CL- Ok. So, what is your experience of anxiety?**

Gillian- Um what do you mean how I feel?

**CL- Yes. What is your experience of it, how you particularly experience in your everyday life if at all?**

Gillian- Don’t know how to answer that um. I get sweaty palms which I think most people do if they’re anxious. I just feel, um, that if we go to the supermarket which I hate, I hate the supermarket. I just um I just wanna get out again you know I don’t feel comfortable in the supermarket um…

It’s worse in the supermarket. I don’t know how to answer that I just feel that I’m just a different person and I’m afraid to go anywhere because I’m afraid I’ll feel ill or something you know….

**CL- How does anxiety affect you? You’ve mentioned sweaty palms, are there any other ways you notice that it affects you?**

Gillian- I don't feel confident to go anywhere on my own. Which it can't believe I’m saying this is it’s so pathetic….

**CL- Do you notice any feelings of anxiety that affect you physically?**

Gillian- I don't think so. No I’m not sure. You’re asking difficult questions ha… Do other people proper answer them properly I don’t really know how to answer it?!

**CL- There’s not a proper answer. The answers are your answers so they are proper answers because they’re yours.**

Gillian- All I can say is I’ve lost my confidence. And I hate the way I feel. I try to avoid people. I don't want to talk to anybody. I don’t mind talking to you but that’s different. I don't like having to say to my friends, I don’t know why they’re still my friends but that would be me, I don't like having to say to them I can't make or I can’t make to this and I hate having to say that you know. I think here we go again I’m letting people down…

**CL- You’ve touched little bit on how some of the ways anxiety can make you feel then, can describe any more how anxiety makes you feel? Are there any particular words you would use of how it makes you feel?**

Gillian- I feel like just want to come back, get home, you know. I feel like I feel safe in my house you know. I’m better when I’m got to do something you know, because when I’ve got nothing to do then I just feel sorry for myself I suppose. And everything’s an effort but when I’ve <mumbling>.

**CL- So how do you react to anxiety?**

Gillian- Gosh I don’t know um. In what situation would you….

**CL- For example if you’re in the supermarket..**

Gillian- I can’t wait to get out.

**CL-Are there any things that you do in response to anxiety to help you overcome that situation?**

Gillian- Blimey you’re asking me really difficult questions here heh. Well I try, I’m a good actress. If I’m out anywhere I can, people wouldn't know that I was feeling the way I feel. I don’t know how to answer that I’m sorry.

**CL- That’s ok.**

Gillian- I don’t know how to answer that question.

**CL- Is there anything else that you think would be helpful for me to know that we’ve not touched upon?**

Gillian- In relation to what?

**CL- In relation to talking about anxiety and Parkinson’s, and-**

Gillian- Well I hate having it. I’d rather not have it. As you can probably imagine other people might say the same I don’t know. But I wish I could be like people that <partner> knows and the people doctor treats. ‘Cos he says they all, well say 99% of them take the medication and feel ok. I’ve have had umpteen blood test done, they all come back normal. Nobody can seem to find anything wrong. Why I’m acting like I am. And I don't know whether that is part of Parkinson’s or what I don't know. I mean I’ve read lots about it and some of it I can relate too you know. But… I don’t know how to answer the question I’m sorry.

**CL- That’s ok. Have you had any experiences with any other types of support other than medication?**

Gillian- No.

**CL- Has that ever been offered to you at all?**

Gillian- Um I know there’s a help group and every time I know it's on I think I’ll go but when it comes to the day I don't do it.

**CL- What’s the reason for not getting to it?**

Gillian- I suppose I can’t be bothered. Basically… and sometimes I think um would I see people a lot worse than me which, I don't know I don't think that would put me off I think it's just the lack of enthusiasm to do it. If I felt better in myself I would yeah. I did ring up about it and found out information. It’s down at <church> which is by <location>…

**CL- I really appreciate the time you’ve taken for this interview. Is there anything else at you can think of that might be helpful for me to know?**

Gillian- Not really no.

**CL- Have you got any questions for me?**

Gillian-…… I don’t know. Um I don't think so. Um no.

**CL- If you do after the interview my mobile number is on the information sheet as well as my e-mail address so if you've got any questions you can always get hold of me again. Have you got any questions for me <partner?>**

Partner- Um yeah how many people are you interviewing?

**CL- So for this study, six. It’s quite a small study and the reason we’re keeping it quite small at the moment because in the work I’ve there was lots of evidence to do with Parkinson’s and anxiety around the numbers. So they know how many people with Parkinson’s experience anxiety, things like that. But there’s not actually any evidence around how people with Parkinson’s experience anxiety. No one’s actually gone and asked them. So we’re fairly confident this is one of the first studies of its type and so this is a very small exploratory study to test the waters with a few people to see what sort of experiences there are out there. And in that way it starts to lay a foundation to build future work upon.** **The aim of that future work is to develop an anxiety intervention for people with Parkinson's that is built from the ground up from their experiences. We’d like to develop something from the ground up with the input of people with Parkinson's and based on their experiences.**

Partner- So you’re interviewing six people and Gillian is number?

**CL-Six**.

Partner- Number six!

**CL- You are number six.**

Partner- Are there any sorts of trend developing?

**CL- I wouldn't want to say in case I have interpreted it wrong at this point. But it does lead on to my next point, I was wondering JM if you'd be interested in me sending you a summary of the findings which would probably answer your question a little bit clearer <partner>.** **So you’d get a summary of the findings before before anything was published or went out.**

Gillian- So is a well-known fact that anxiety and depression are part of Parkinson’s?

**CL- Yes.**

Gillian- Because I’ve read that it is.

**CL- There is a lot of evidence to support that anxiety and depression are very common, what are called non-motor symptoms of Parkinson's. There were some big studies that found 98% of people with Parkinson experienced non-motor symptoms. That can be anything from tummy troubles, sleeping problems, memory problems, pretty much most things that aren’t to do with movement.** **And of that 98% around half of those people, the precise number is about 56.4%, experience anxiety. So it’s a very very high number of the population of people with Parkinson’s experience anxiety.**

Gillian- So I’m not unique ha.

**CL- I think your experience is shared by lots of people. You’re certainly not alone in what you experience. Even though I’m sure it feels very much like that.**

Gillian- Oh it does yeah. It’s horrible. Like I said if you knew me before this I was totally totally different. I feel like I've just changed to somebody I don't like you know. I hate it. And we’re getting older and you don’t know how much time you’ve got left do you. You get to 73. I can’t believe I’m saying I’m 73 either. But I’m glad maybe other people feel the same then.

**CL- The findings I will send you, the summary, I'm very aware that my interpretation of them, when I'm analysing them with some other researchers, I’m aware that none of us are people with Parkinson's. So you to make sure we are not interpreting them in our way I'm asking if all of the participants would be happy to give comments on them to make sure they are accurate and we’re portraying your experiences in the right way. Would you be happy to do that?**

Gillian- Mmm.

**CL- It wouldn’t be very labour-intensive it would be you reading over the summary and telling me if you were happy with it or not. Would you prefer me to send that to you by post or by email?**

Gillian- Yeah you can send it <partner> e mail.

Partner- Can I ask when you release these findings will JM be anonymous?

**CL- Yes you will be anonymous. So I’m making this recording on an encrypted Dictaphone. I then download it to an encrypted computer. I myself will make a transcript of it and in that process I will you give you a pseudonym and also <partner> you will be anonymised as well. Once I've done that the actual files will be electronically shredded once I finished writing those transcripts up. So it's as if that never existed there’s no way of ever getting that back. After the study all of the information will be stored securely. Everything will be totally anonymous. I tend to send a letter to peoples GPs telling them you’ve taken part in a research study. It's a standardised letter. It doesn't detail anything you told me today. It just says that you've been in a research study and if the GP has any questions they can get in contact with me.**

Gillian- Mmm yeah that’s ok.

**CL-I’ll take your GPs details after this recording. Have you got any other questions for me at all?**

Gillian- I don’t think so no.

Partner- No.

**CL- Thank you very much for your time. I will stop the recording now.** <recording stops>
