# Supplementary material for: What is the lived experience of anxiety for people with Parkinson’s? A phenomenological study
Source: PLoS One. 2021 Apr 8;16(4):e0249390. doi: 10.1371/journal.pone.0249390 (PMC8031398; doi:10.1371/journal.pone.0249390)
Supplement: Attachment — Submitted filename: Response to reviewers.docx [file pone.0249390.s011.docx]

Dear Rosemary Frey,

PONE-D-20-03238R2
What is the lived experience of anxiety for people with Parkinson’s? A phenomenological study
PLOS ONE

Please find attached our revised manuscript as a separate file labelled 'Revised Manuscript with Track Changes'. We are grateful to the reviewers for their comments, particularly their positive encouragement to revise the draft, and have summarised our responses in the table below.

| **Reviewers' comments** | **Responses to Reviewers** |
| --- | --- |
| Reviewer #2: I think the authors have dealt well with the comments from both reviewers. I don’t think there is any need to make further changes on the issues they requested clarity on. The amendments have improved the paper and improved the transparency of the phenomenological method. | Thank you for the feedback. It is gratifying to know we have addressed your comments. |
| Reviewer #3: I believe this study is indeed an important contribution to the phenomenological understanding of Parkinson’s disease. I find its ability to connect what can sometimes be an abstract field of study to clinical intervention as an important example for future work. | Thank you for this feedback which was helpful in preparing the revised manuscript. |
| Reviewer #3: I do believe that the study would benefit from further refinement – particularly as it concerns some of the study’s philosophical and technical positions.  My main concern is with the study’s description of its methodology. I am unsure if the reader can understand it as a cohesive whole and if it is conveyed in a way that would allow future researchers to replicate the study’s methodology. | We have tried to address each of the comments identified and hope that in doing so we have made the methodology more cohesive and promoted the replicability of the study. |
| Reviewer #3: a. The study cites several sources from which it derives aspects of its methodology. However, the borrowed components are left vague. Assuming that you are using a somewhat novel approach (i.e., not “one” comprehensive model like the “Phenomenological Psychological Method [Giorgi, & Giorgi, 2003], the Interpretive Phenomenological Analysis [Smith, & Shinebourne, 2012], etc.), the reader requires further explanation regarding rational and execution of these different aspects. This can be done similarly to how the author explained how the thematic analysis was conducted. | You are right we did not use one definitive approach to phenomenology and drew on a number of sources to inform our methodology. We hope the amendments we have made to different aspects highlighted means the text is more cohesive. We appreciate your openness to how we have approached phenomenology. |
| Reviewer #3: The following questions should be taken into consideration – for most a sentence or two should suffice: i. How does one “bracket” according to Chan et al? (pg. 6) You name some of the steps in the process, but the reader is not sure how you implemented them or what they necessarily mean. | We have annotated the text with explanations of Chan et al’s approach and explicitly stated that these steps were used in the study. |
| Reviewer #3: ii. How did you use Bevan to create the interview? Or what suggestions did you follow? (pg. 8) | We have amended the text to explain that we adapted the wording with an example to bring this alive for the reader. |
| Reviewer #3: iii. What is the “immersion phase” and where does it come from? (pg. 9) | We have added in a succinct definition with a reference to the literature so it evident to the reader where the concept comes from. |
| Reviewer #3: iv. You mention using NVivo v10 – what does the software do? Some readers will be unfamiliar with this. (pg. 9) | We have added in a definition for anyone unfamiliar with this software. |
| Reviewer #3: v. Where does the term “Trustworthiness” come from? (pg. 9) Is this a technical term or did you coin it? This seems similar to the hermeneutic circle (See comments below re: hermeneutics). | We have defined this and added in a reference. |
| Reviewer #3: vi. Lastly, perhaps it is worth the authors mentioning how the above techniques fit together under a descriptive phenomenological framework. Or perhaps brief elaborations on the above-mentioned points will make this self-evident. | We hope the way we have elaborated on the text has made this self-evident. |
| Reviewer #3: 2) Another concern is some ambiguity or confusion on study’s position as representing “descriptive phenomenology.” The study seems to claim to be descriptive (i.e. presenting a phenomenon without using foreknowledge or interpretation) while also offering some of the author’s interpretations and contextualization, which is more akin to a hermeneutic or interpretive stance. | As with point one, we hope the amendments we have made to different aspects highlighted mean it is much clearer that this is a descriptive phenomenology. |
| Reviewer #3: The following are several points that warrant attention: a. If this study is to be considered a “descriptive phenomenology” the authors should seriously consider separating out the descriptions of the themes they found from any interpretations of such themes. | This has been done. |
| Reviewer #3: Some instances include: i. The use of psychological concepts of “upward and downward comparison” to explicate coping mechanisms. (pg. 12) | As well as separating the findings and discussion, the text is amended to show what was described by the participants and how it relates to the existing literature. |
| Reviewer #3: ii. Offering explanation of adverse social experiences via cognitive psychology (i.e. “slowed cognitive processing”). (pg. 17) This might be remedied by tying this theme to your findings that subject report “inability to think,” as opposed to the author’s cognitive explanation. |  |
| Reviewer #3: iii. Interpreting the theme of “Ability to think” through a cognitive explanation (i.e. overload of working memory). (pg. 18) | The text has been reordered to resolve this confusion (see p26 in revised manuscript). |
| Reviewer #3: iv. Interpreting emotion experience through physiological framework (i.e., dopaminergic pathways). (pg. 19) | The text has been reordered to resolve this confusion (see p26 in revised manuscript). |
| Reviewer #3: v. Introducing powerlessness/meaninglessness to extrapolate when discussing subjects’ experience of loss of control. (pg. 26) This does not seem to come directly from subject reports. | We have moved this point to the discussion section and reworded to add clarity. |
| Reviewer #3: vi. Using pathophysiology to explain omnipresent anxiety. (pg. 29) | We have linked this back to our introductory literature review in a way that we hope adds clarity. |
| Reviewer #3: b. One possible solution to the issue above is for the authors to separate out the interpretations of subjects’ accounts from the accounts themselves – possibly by differentiating “Results” section (i.e. descriptions of subject reports) from a “Discussion” section (i.e. elaborations, interpretations, and comparison of subject accounts to other fields of study or established work). I see that another reviewer suggested a similar edit, but I hope these points illuminate some of the theoretical reasons why one might consider doing so. To clarify, I believe these interpretations and comparisons are useful and have good insight, but to be truly “descriptive” one should rely more purely on subject reports. | We have done this. We did not fully understand the import of what the previous reviewer was suggesting. Thanks you for the additional clarification here. |
| Reviewer #3: c. In addition, it should be pointed out that the term “lifeworld,” which the study uses, is a term borrowed from hermeneutic, or interpretive phenomenology. The term is an effort by Heidegger to emphasize the need to contextualize a person’s experience, which is opposed to the aspirations of Husserl’s bracketing. This is not to say that the authors should do away with this word, as it has been widely adopted throughout phenomenological research, but rather it brings attention to some ambiguity in the study’s philosophical underpinnings. | We appreciate your observation and acknowledge the ambiguity that could arise but have kept the concept in. This is because it was a recommendation in a previous review and we believe it does enhance the understanding of the lived experiences of the participants in this study. |
| Reviewer #3: d. For further overview on the difference between descriptive and interpretive phenomenology, I suggest looking at Lopez & Willis’ “Descriptive versus interpretive phenomenology: Their contributions to nursing knowledge” (2004). | Thank you for this suggestion and we have incorporated this reference into the text. |
| Reviewer #3: 3) The study overall would also benefit from further copy editing and general tightening up some of the prose throughout the paper. Two examples below: | We have tried to copy edit and tighten up the prose. |
| Reviewer #3: a. Is the following a statement or a question? --- “Therefore, perhaps a shift in perspective is required so that healthcare professionals and researchers are more aware of the priorities of PWPs?” (pg. 23) | We have edited the text so it now reads –  “Therefore, a shift in perspective may be required so that healthcare professionals and researchers are more aware of the priorities of PWPs.” |
| Reviewer #3: b. This sentence could benefit from a comma after “insights” and perhaps hyphenating “anxiety-specific” --- “Whilst these studies provide insights generally there is a lack of anxiety specific research in Parkinson’s.” (pg. 5) | We agree that this improves the sense of the sentence and have made the edits suggested. |
| Reviewer #3: Overall, I think this is a valuable piece of work. I hope you will consider the above revisions. There is indeed no one way to conduct phenomenology, but I believe taking these points into consideration will improve the study’s philosophical and methodological groundings. | Thank you for being so encouraging. We hope the resultant edits have improved the study’s philosophical and methodological groundings. |

We hope we have addressed the comments sufficiently in the revised manuscript to merit publication and we look forward to hearing from you in due course.

Kind regards,

Chris Lovegrove

Katrina Bannigan
